# The role of SIRT1 in autophagy and drug resistance: unveiling new targets and potential biomarkers in cancer therapy

**DOI:** 10.3389/fphar.2024.1469830

**Published:** 2024-09-30

**Authors:** Yujing Tang, Wantao Ju, Yanjun Liu, Qin Deng

**Affiliations:** ^1^ School of Life Science and Engineering, Southwest Jiaotong University, Chengdu, China; ^2^ Obesity and Metabolism Medicine-Engineering Integration Laboratory, Department of General Surgery, The Third People’s Hospital of Chengdu, Affiliated Hospital of Southwest Jiaotong University, Chengdu, Sichuan, China; ^3^ Department of Breast and Thyroid Surgery, The Second Affiliated Hospital of Chongqing Medical University, Chongqing, China

**Keywords:** autophagy, apoptosis, ferroptosis, drug resistance, sirtuin family, SIRT1

## Abstract

Cancer, the world’s second leading cause of death after cardiovascular diseases, is characterized by hallmarks such as uncontrolled cell growth, metastasis, angiogenesis, hypoxia, and resistance to therapy. Autophagy, a cellular process that can both support and inhibit cancer progression, plays a critical role in cancer development and progression. This process involves the formation of autophagosomes that ultimately fuse with lysosomes to degrade cellular components. A key regulator of this process is Sirtuin 1 (SIRT1), which significantly influences autophagy. This review delves into the role of SIRT1 in modulating autophagy and its broader impacts on carcinogenesis. SIRT1 regulates crucial autophagy mediators, such as AMP-activated protein kinase (AMPK) and mammalian target of rapamycin (mTOR), effectively promoting or suppressing autophagy. Beyond its direct effects on autophagy, SIRT1’s regulatory actions extend to other cell death processes, including apoptosis and ferroptosis, thereby influencing tumor cell proliferation, metastasis, and chemotherapy responses. These insights underscore the complex interplay between SIRT1 and autophagy, with significant implications for cancer therapy. Targeting SIRT1 and its associated pathways presents a promising strategy to manipulate autophagy in cancer treatment. This review underscores the potential of SIRT1 as a therapeutic target, opening new avenues for enhancing cancer treatment efficacy.

## Graphical Abstract

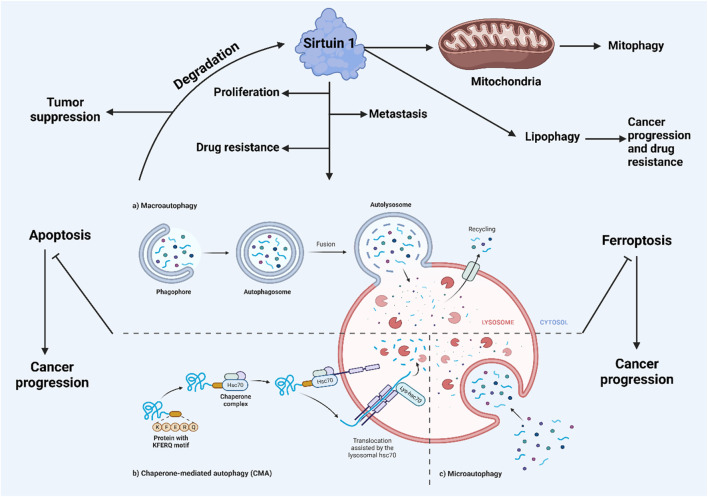

## Highlights

• As a cell death mechanism, autophagy regulates initiation and progression of carcinogenesis.
• Sirutin family has various cellular functions in which SIRT1 is the most well-known one.
• SIRT1 modulates autophagy and other selective types including mitophagy and lipophagy.
• SIRT-mediated autophagy can regulate apoptosis occurrence in tumor cells.
• SIRT1-mediated autophagy regulation determines the response to cancer chemotherapy.

## 1 Introduction

Cells utilize autophagy and the ubiquitin-proteasome degradation pathway to dispose of toxic, misfolded, damaged, or unnecessary proteins (Wirawan et al., 2012). Unlike the proteasome, autophagy can degrade a vast array of substrates, including large protein aggregates and entire organelles. Beyond proteins, autophagy also breaks down lipids, DNA, and nuclear RNA, generating new pools of amino acids, fatty acids, and nucleosides for use in anabolic processes. This continual turnover facilitates a cycle of cellular breakdown and renewal (Rabinowitz and White, 2010). Autophagic degradation is carried out by lysosomes, which contain acidic hydrolases such as peptidases, lipases, and nucleases, breaking down large molecules into simpler components. Although all autophagic pathways converge at the lysosomal compartment (or vacuole in yeast), several routes exist to reach these lysosomes. In mammalian cells, three primary autophagy processes are recognized: chaperone-mediated autophagy (CMA), microautophagy, and macroautophagy (Cuervo, 2004). CMA targets proteins with a KFERQ-like motif to the lysosomes, facilitated by heat shock cognate 70 and its co-chaperones, with the lysosomal-associated membrane protein 2 (LAMP-2A) (Kaushik et al., 2011) mediating their subsequent breakdown. Microautophagy involves the lysosomal membrane invaginating to engulf cytoplasmic material, which is then degraded. Macroautophagy, on the other hand, involves the formation of autophagic vacuoles through the creation of autophagic membranes (phagophores) that evolve into double-membraned vesicles called autophagosomes. This form of autophagy is evolutionarily conserved across all eukaryotic cells and has been extensively studied, particularly through mouse models focusing on macroautophagy.

Cancer remains one of the most prevalent diseases globally, irrespective of economic status, with approximately 18.1 million new cases and 9.1 million deaths reported in 2018 (Bray et al., 2018). The extensive research over the past decades into cancer development, progression, detection, and treatment has highlighted the critical nature of early diagnosis and intervention. Without these, cancer often proves fatal. Despite significant advancements, cancer multidrug resistance continues to be a significant obstacle in effective cancer treatment. Chemotherapy remains a cornerstone for treating various malignancies across different stages. Researchers often grapple with understanding the development and potential treatments of cancer, not foreseeing the emergence of drug resistance within their studies. Drug resistance in cancer is complex and broad, making it a challenging phenomenon to elucidate. The understanding of chemotherapeutic resistance mechanisms has expanded greatly, yet the scientific explanations remain limited. The strategies by which tumor cells manage their metabolic pathways and signaling can influence treatment outcomes, such as preventing drug penetration into cancer cells and promoting drug efflux. Numerous studies have also explored whether specific genes are upregulated to foster drug resistance, examining aspects like drug transport through tumor cells, membrane transport protein pathways, target molecule overexpression, direct gene transcription, anti-apoptosis, and enhanced DNA repair, all of which have been implicated in the promotion of drug resistance (Harguindey et al., 2005; Housman et al., 2014; Liang Z. et al., 2014).

**FIGURE 1:**
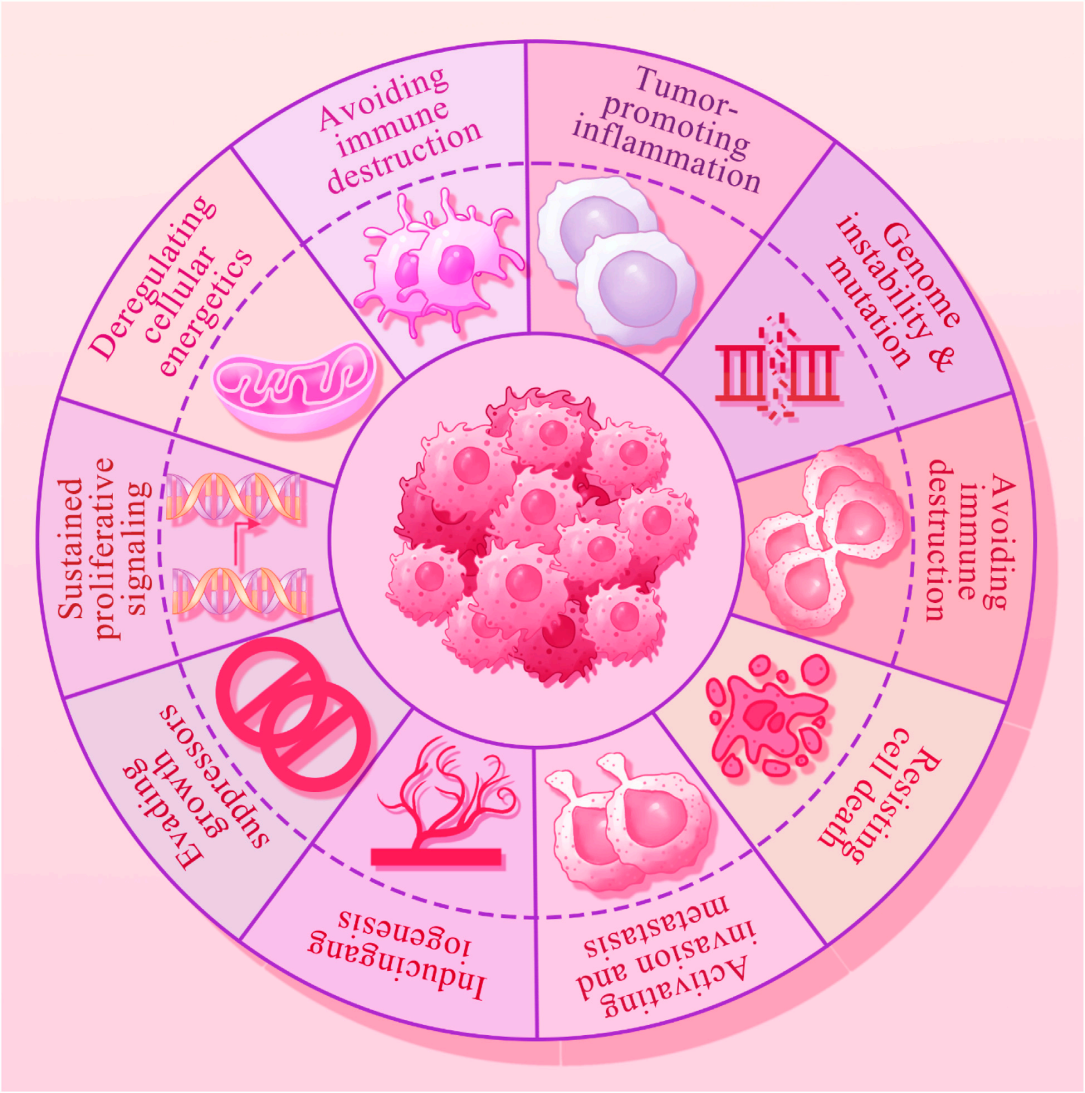
The hallmarks of cancer that include the immune escape, epigenetic alterations, oncogenic inflammation, genomic instability, increased proliferation, angiogenesis, metastasis, cell death resistance and replicative immortality.

Dysregulation of cell death mechanisms is a common feature in carcinogenesis (Figure 1), influencing tumor cell survival, viability, proliferation, metastasis, and response to therapy. Autophagy, a cellular catabolic process, involves the breakdown and recycling of proteins and organelles. It starts with the formation of an autophagosome, a vesicle that fuses with a lysosome containing hydrolytic enzymes. Unlike mitophagy, which specifically targets intracellular organelles, macroautophagy is a non-selective form of autophagy. The complex molecular process of autophagy, which includes nucleation, elongation, and fusion, is facilitated by various proteins, including the autophagy-related (ATG) protein family (Ferro et al., 2020; Ichimura et al., 2000; Mizushima et al., 1998; Zaffagnini and Martens, 2016). Autophagy plays a critical role in balancing environmental substrate availability with cellular metabolic demands. It is activated by nutrient deprivation and oxidative stress through well-regulated pathways linked to energy metabolism, involving key regulators such as mTORC1 and AMPK (Hosokawa et al., 2009; Jung et al., 2009; Egan et al., 2011; Kim et al., 2011). The two primary physiological roles of autophagy are the degradation of defective proteins or organelles for quality control and the recycling of macromolecules under conditions of nutritional stress to meet metabolic needs (Kim and Lee, 2014; Mizushima and Komatsu, 2011).

In recent years, research has increasingly focused on the role of autophagy in both physiological and pathological contexts. Autophagy serves a dual and crucial role in cancer, where it can either promote or inhibit tumorigenesis. While commonly recognized as a mechanism of cell death, autophagy also influences metastasis and resistance to therapy (Qin et al., 2023; Ashrafizadeh et al., 2022). Consequently, it has become a promising target for pharmacological compounds and nanoparticles in cancer treatment (Ashrafizadeh et al., 2020a; Paskeh et al., 2022). With a better understanding of various autophagy regulators now available, this review concentrates on the role of SIRT1 in autophagy regulation within human cancers. It explores SIRT1’s association with cancer hallmarks and its interactions with apoptosis and ferroptosis.

## 2 A history of cell death

Cell death is essential for eliminating undesirable or damaged cells, playing a crucial role in animal development, tissue homeostasis, and stress response (Chen et al., 2016). Improper regulation of cell death contributes to various human diseases, including cancer and inflammatory disorders. Oncogenic transformation allows neoplastic cells to develop resistance to cell death, aiding their survival and the accumulation of mutations that promote cancer development (Hanahan and Weinberg, 2011). Many chemotherapeutic drugs work by inducing cell death, making it a fundamental strategy in cancer treatment. Consequently, targeting cell death mechanisms offers a promising approach for developing new anticancer drugs. Cell death can be classified based on morphological and biochemical characteristics into several primary types, such as apoptosis, necrosis, autophagic death, and mitotic catastrophe (Galluzzi et al., 2012). Historically, necrosis was considered a passive and uncontrolled process, while apoptosis was understood as a highly regulated, programmed cell death. However, the past 2 decades of research have revised this view, revealing a regulated form of necrosis. It was discovered that in some cells, inhibiting caspases, which are crucial for apoptosis, did not stop cell death but instead shifted it towards necrotic symptoms (Vercammen et al., 1998). Further studies identified receptor-interacting kinase 1 (RIP1; RIPK1) as a key regulator of this form of necrosis (Holler et al., 2000). Chemical biology research led to the identification of small-molecule inhibitors targeting this cell death pathway (Degterev et al., 2005), specifically inhibiting RIP1 (Degterev et al., 2008). Recent studies have established that RIP3 acts as a downstream mediator of RIP1 (Cho et al., 2009; He et al., 2009; Zhang et al., 2009), with the Mixed Lineage Kinase Domain-Like (MLKL) protein playing a central role in executing cell death (Sun et al., 2012). The physiological and clinical relevance of necrosis has been underscored by various studies in living organisms (Belizário et al., 2015). This regulated form of cell death, now termed necroptosis, involves RIP1, RIP3, and MLKL (Galluzzi et al., 2012) and is essential for its execution. Increasing evidence suggests that necroptosis acts as a protective mechanism by eliminating cancer cells that are resistant to apoptosis, highlighting its significant role in both the biology and therapy of cancer. Table 1 summarizes the dysregulation of cell death mechanisms in human cancers.

**TABLE 1:** A summary of dysregulated cell death mechanisms in tumors.

Cell death	Cancer	Remark	Reference
Ferroptosis	Colorectal cancer	This study identifies METTL17 as a key regulator of mitochondrial function and ferroptosis resistance in CRC, showing that its depletion sensitizes CRC cells to ferroptosis and inhibits tumor growth	Li et al. (2024a)
Ferroptosis	-	USP8 stabilizes GPX4 to counteract ferroptosis, and its inhibition sensitizes cancer cells to ferroptosis	Li et al. (2024b)
Ferroptosis	Breast cancer	This study identifies Acod1 as a key metabolic enzyme in tumor-infiltrating neutrophils (TINs) that protects them from ferroptosis and promotes metastasis	Zhao et al. (2023a)
Autophagy	-	YY1 promotes gastric cancer progression by enhancing autophagy through ATG4B transactivation, and is regulated by ALKBH5 and YTHDF1 via m6A modification	Wang et al. (2023a)
Autophagy	Colon cancer	KLF4 suppresses 5-FU resistance in colon cancer cells by inhibiting autophagy through targeting RAB26, and its overexpression reduces proliferation and drug resistance	Zheng et al. (2023)
Autophagy	Pancreatic cancer	The fructose metabolism, mediated by GLUT5, supports pancreatic ductal adenocarcinoma (PDAC) progression by enhancing cell survival, proliferation, and metabolic plasticity, while inhibiting autophagic cell death through the AMPK-mTORC1 pathway	Cui et al. (2023)
Autophagy	Bladder cancer	The loperamide inhibits bladder cancer cell proliferation by inducing autophagy and apoptosis through the ROS-mediated JNK pathway, and combining loperamide with autophagy inhibitor CQ enhances its anti-cancer effects	Wu et al. (2023)
Autophagy	Pancreatic cancer	Inhibiting CAF autophagy suppresses tumor development and enhances anti-tumor immunity by reducing CD274/PDL1 expression in PDAC, with targeted CAF autophagy inhibition via chloroquine diphosphate-loaded MSC-liposomes improving immunochemotherapy efficacy	Zhang et al. (2024a)
Autophagy	B-cell malignancy	The cancer-intrinsic autophagy, involving key genes like ATG3, BECN1, and RB1CC1, protects tumor cells from CD19 CAR-T cell-mediated cytotoxicity, and its inhibition sensitizes B-cell leukemia and lymphoma cells to CAR-T therapy	Tang et al. (2024)
Apoptosis	Gastric cancer	DHRS4-AS1 is significantly downregulated in GC, inhibiting GC cell proliferation and promoting apoptosis by degrading the pro-oncogenic DHX9 and disrupting the DHX9-ILF3 interaction that activates NF-kB signalling	Xiao et al. (2023)
Apoptosis	Pancreatic cancer	This study introduces CK21, a novel pro-drug of triptolide, which demonstrates potent anti-proliferative effects on pancreatic cancer by inhibiting the NF-κB pathway, increasing oxidative stress, and inducing mitochondrial-mediated apoptosis, while showing minimal toxicity	Tian et al. (2023)
Apoptosis	Cervical cancer	The galectin-7 enhances cisplatin-induced apoptosis in cervical cancer by promoting mitochondrial dysfunction and ROS generation, while reducing chemoresistance by facilitating stress granule clearance via the galectin-7/RACK1/G3BP1 axis	Liu et al. (2023)
Apoptosis	Colorectal cancer	5-MTP promotes apoptosis, induces cell cycle arrest, and inhibits cell proliferation in colorectal cancer (CRC) cells, with these effects significantly enhanced when combined with PI3K/Akt/FoxO3a signaling pathway inhibitors	Zhao et al. (2023b)
Apoptosis	Ovarian cancer	METTL3 is highly expressed in EOC and promotes cell proliferation, while its silencing induces cell cycle arrest and apoptosis through the FAS/FADD and mitochondrial pathways. Sulfuretin (Sul) enhances apoptosis in EOC cells by downregulating METTL3 and reversing the effects of METTL3 overexpression	Yu et al. (2023a)
Apoptosis Ferroptosis	Hepatocellular carcinoma	Celastrol (Cel) targets VDAC2 to induce mitochondria-dependent apoptosis and ROS-mediated ferroptosis in hepatocellular carcinoma (HCC), while its encapsulation in alkyl glucoside-modified liposomes (AGCL) enhances its anti-tumor efficacy and reduces side effects	Luo et al. (2023a)
Apoptosis Ferroptosis	Colorectal cancer	CAPG is significantly overexpressed in CRC and correlates with poor prognosis, while its knockdown inhibits CRC cell growth, induces cell cycle arrest, and promotes apoptosis and ferroptosis via the upregulation of the P53 pathway	Zhao et al. (2023c)
Apoptosis	Gastric cancer	TRIM17 is upregulated in GC and promotes tumor cell proliferation and survival by ubiquitinating and degrading BAX, thereby inhibiting BAX-dependent apoptosis	Shen et al. (2023)
Ferroptosis	Cervical cancer	Matrine inhibits tumor growth and induces ferroptosis in SiHa cells by reducing GPX4 levels and increasing intracellular Fe2+, ROS, and lipid peroxides, while upregulating Piezo1 expression and promoting calcium influx	Jin et al. (2024)
Ferroptosis	Breast cancer	TAM resistance in breast cancer is driven by RelB, which inhibits TAM-induced ferroptosis by upregulating GPX4, and that reducing RelB or GPX4 levels can resensitize TAM-resistant cells by promoting ferroptosis	Xu et al. (2023a)
Ferroptosis	Colorectal cancer	The drug-resistant colorectal cancer organoids exhibit elevated LGR4 expression and Wnt signaling activation, which confer resistance by upregulating SLC7A11 to inhibit ferroptosis. Targeting LGR4 with a monoclonal antibody (LGR4-mAb) sensitizes cancer cells to chemotherapy-induced ferroptosis	Zheng et al. (2024a)
Ferroptosis	-	GSTP1 provides a GPX4- and FSP1-independent defense against ferroptosis by detoxifying lipid hydroperoxides, and its degradation via the SMURF2/GSTP1 axis sensitizes cancer cells to ferroptosis-inducing drugs and immune checkpoint inhibitors	Zhang et al. (2023a)
Ferroptosis	Colorectal cancer	ATF3-CBS signaling axis as a key mechanism that enables colorectal cancer cells to evade ferroptosis under cystine deprivation by regulating the mitochondrial TCA cycle. Blocking this axis sensitizes cancer cells to ferroptosis	Liu et al. (2024a)

## 3 Different types of autophagy machinery

### 3.1 Macroautophagy

Macroautophagy, the most extensively examined type of autophagy, plays a crucial role in the breakdown and recycling of cellular components. This process is advantageous in numerous diseases, such as the removal of protein aggregates found in neurodegenerative disorders. Furthermore, macroautophagy has been recognized as a potential therapeutic target in cancer treatment, with its effectiveness depending on the stage of the tumor, its biological characteristics, and the tumor’s microenvironment (Debnath et al., 2023). Autophagosome formation, which involves creating a double-membrane vesicle, is the first step in autophagosomal vesicle generation. These autophagosomes, containing a variety of ATG products, subsequently merge with lysosomes. Lysosomal hydrolases then degrade the autophagosome’s contents. Key protein kinases, ULK1 and ULK2, along with their subunits FIP200, ATG13, and ATG101, initiate autophagosome formation in response to nutritional and energy signals, primarily from mTORC1 signaling. The recruitment of ATG7 and ATG3 is essential when phosphatidylinositol 3-phosphate is activated, facilitating the production of PS3P on autophagic membranes by the specialized Vps34 complex I, which includes Vps34, Beclin-1, ATG14, and Vps15. This complex is vital for cargo recruitment and autophagosome maturation (Zhao et al., 2021; Nakatogawa, 2020). Members of the ATG8 family, divided into two human subfamilies (microtubule-associated protein 1A/1B-light chain 3 (LC3) and GABARAP), are involved in lipid conjugation produced by the Vps34 complex I. Macroautophagy can non-selectively incorporate various materials into autophagosomes, especially under conditions of nutrient scarcity, thereby recycling essential molecules like lipids and amino acids. Consequently, macroautophagy is segmented into four phases: initiation, autophagosome formation, elongation, and fusion of the autophagosome with lysosomes, with each stage meticulously controlled. The final step, fusion, is mediated by SNARE proteins that facilitate the merging with the lysosome.

### 3.2 Microautophagy

Microautophagy includes two forms: selective and non-selective (Wang L. et al., 2023). Similarly, macroautophagy can also engage in either selective or non-selective absorption and degradation of cargoes. The cellular context influences whether microautophagy targets specific cargoes or functions non-selectively (Mijaljica et al., 2011). Historical studies primarily focused on microautophagy in rat liver before the discovery of ATG genes. In these studies, rat liver lysosomes were observed to invaginate their membranes and engulf various cargoes such as hemoglobin, ovalbumin, lysozyme, ferritin, and Percoll particles, facilitated by their acidic internal pH (Ahlberg et al., 1982; Marzella et al., 1981; Ahlberg and Glaumann, 1985). Certain drugs known as lysosomotropic agents, such as chloroquine, can inhibit the breakdown of these materials within the lysosomes. Findings indicate that the main autophagic response to starvation and refeeding in mice and rat livers is microautophagy (de Waal et al., 1986; Mortimore et al., 1988; Mortimore et al., 1983). However, these studies primarily utilized electron microscopy to observe morphological changes and lacked detailed biochemical evidence of alterations in autophagic activity or the molecular pathways involved. Macroautophagy can selectively target specific cargoes based on environmental conditions. Various selective forms of macroautophagy have been identified, including xenophagy for microorganisms, aggregephagy for protein aggregates, mitophagy for mitochondria, reticulophagy for the endoplasmic reticulum, lysophagy for lysosomes, and ribophagy for ribosomes (Kirkin and Rogov, 2019; Anding and Baehrecke, 2017). Recent research has also highlighted different types of selective microautophagy such as endosomal microautophagy (eMI), micronucleophagy, and micromitophagy, each believed to be regulated by distinct molecular pathways and serving unique functions.

While the direct role of microautophagy in cancer progression modulation has been overlooked, the pathways it influences are better understood (Wang L. et al., 2023). The Wnt signaling pathway regulates various biological processes including development, self-renewal, and immune surveillance (Galluzzi et al., 2019; Nusse and Clevers, 2017). Inhibition of GSK3 triggers the Wnt pathway, and microproteophagy contributes to the degradation of GSK3 and its associated substrate, SMAD4 (Albrecht et al., 2018; Taelman et al., 2010). The degradation of GSK3 by Wnt, facilitated through microproteophagy, depends on the availability of methionine (Albrecht et al., 2019). This establishes a link between microautophagy’s role and the regulation of pathways that influence the proliferation and survival of cancer cells. Furthermore, tumor cells may utilize MDV-induced micromitophagy to enhance their adaptability and survival, underscoring that targeting both macromitophagy and micromitophagy could enhance the efficacy of cancer therapies (Towers et al., 2021).

### 3.3 CMA

**FIGURE 2:**
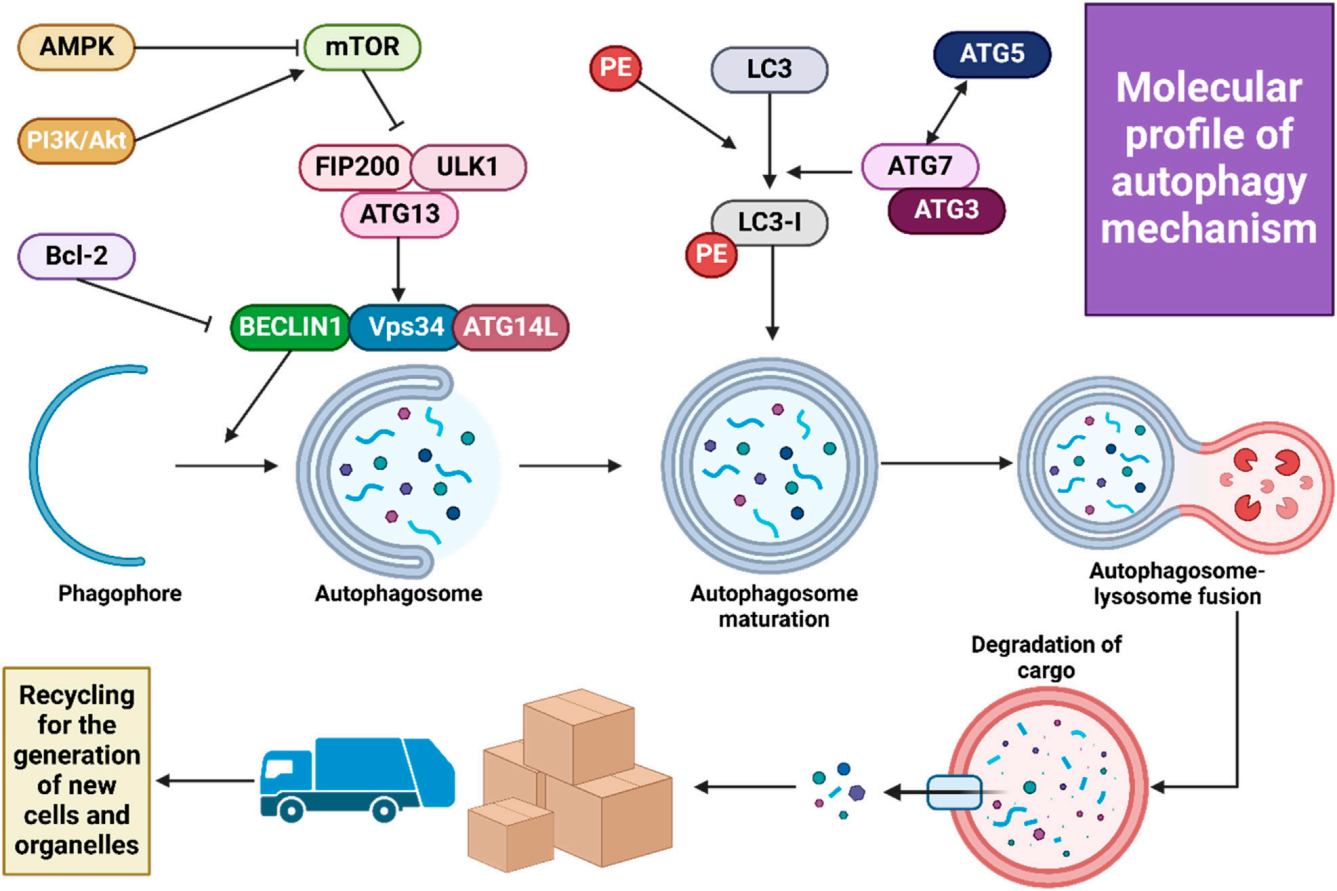
A schematic illustration of autophagy mechanism.

Three types of intracellular lysosomal degradation and autophagy exist, among which CMA is one (Assaye and Gizaw, 2022). CMA specifically targets proteins that are damaged or abnormal for degradation. It distinguishes itself from the other two types of autophagic processes in two keyways. Firstly, it uniquely requires the specific translocation of cargo proteins directly across the lysosomal membrane without enclosing them in a vesicle, allowing these proteins to enter directly into the lysosomal lumen (Auzmendi-Iriarte and Matheu, 2020). Secondly, CMA selectively degrades specific proteins from a larger pool, facilitated by a recognition motif similar to KFERQ found in proteins it targets (Xilouri and Stefanis, 2015). This selectivity enables CMA to degrade only the damaged or abnormal proteins without affecting the normal proteins, even if these are part of a multi-protein complex (Cuervo and Wong, 2014). Furthermore, CMA plays a crucial role in regulating various cellular processes by influencing levels of intracellular enzymes, transcription factors, and cell maintenance proteins. This impacts proteostasis, cellular energetics, and immune system functionality, depending on which proteins are selected for degradation at any given time (Auzmendi-Iriarte and Matheu, 2020; Cuervo and Wong, 2014). Figure 2 illustrates the macroautophagy mechanism.

The role of CMA in cancer therapy has garnered attention. For instance, FDW028 has been found to inhibit lysosomal proteolysis via CMA, which in turn can hinder metastasis in colorectal cancer (Wang M. et al., 2023). Additionally, CMA-mediated degradation of Dicer has been linked to increased metastasis in breast cancer cells (Su CM. et al., 2023). These findings suggest that CMA’s function extends beyond promoting cell death; it also plays a critical role in regulating metastasis and invasion in cancer cells. In prostate cancer, the protein TPD52 has been observed to activate CMA through its interaction with HSPA8/HSC70, leading to enhanced substrate degradation. The upregulation of TPD52 is crucial for promoting growth and stress resistance in prostate cancer cells (Fan et al., 2021). Beyond influencing metastasis, CMA is implicated in regulating growth and drug resistance in various cancers (Ichikawa et al., 2020). Furthermore, CMA is capable of degrading IGF-1Rβ in pancreatic cancer, demonstrating its impact on other significant factors (Xue et al., 2019).

## 4 Autophagy machinery function in oncology

Studies involving cell cultures and pre-clinical animal models have demonstrated that autophagy, along with genome integrity and anti-inflammatory signaling pathways, plays a crucial role in maintaining tissue homeostasis and preventing pro-oncogenic conditions (Amaravadi et al., 2016; Mathew et al., 2009; Long and McWilliams, 2020). Although there are instances of polymorphisms and altered expression levels of ATG proteins, key autophagy genes are generally unmutated in human cancers (Jiang and Mizushima, 2014). Additionally, autophagy genes are associated with either promoting or inhibiting tumor growth (White, 2015). The discovery of frequent loss of the autophagy regulator Beclin-1 (BECN1) in many cases of human breast, ovarian, and prostate cancers has shed light on the role of autophagy in oncology, suggesting that BECN1 may act as a tumor suppressor gene, particularly in individuals with only one functional copy (Yue et al., 2003; Qu et al., 2003; Karantza-Wadsworth et al., 2007). This theory is supported by findings in heterozygote Becn1 mice, which exhibit an elevated risk of developing hepatic, breast, and lymphoid tumors (Karantza-Wadsworth et al., 2007). While the status of BECN1 as a *bona fide* tumor suppressor remains under debate, its significant cellular role is undeniable (Li et al., 2017). In recent research, scientists created knock-in mice with a constitutively active Beclin-1 variant (Becn1F121A/F121A) that disrupts the interaction between endogenous Beclin-1 and its inhibitor Bcl-2. This alteration led to increased autophagic activity, improved overall health, extended lifespan, and a lower incidence of age-related spontaneous cancers in these mice (Fernández et al., 2018).

Research has identified patterns of overstimulated, understimulated, and deregulated autophagy (Ozpolat and Benbrook, 2015). The role of autophagy in cancer—whether it is oncogenic or tumor-suppressing—is still a subject of debate (Kroemer and Jäättelä, 2005; Ogier-Denis and Codogno, 2003; Scott et al., 2007; Dalby et al., 2010; Golstein and Kroemer, 2007; Tóth et al., 2002). Autophagy in cancer cells is influenced by various cellular factors, including gene mutations, abnormalities, the activation or inactivation of signaling pathways, and the level of cellular stress. Cancer cells often exhibit a higher rate of autophagy compared to normal cells, which can accelerate their proliferation. For instance, while normal breast epithelial cells typically display high levels of the Beclin-1 protein, these levels are significantly reduced or absent in breast cancer cells (Liang et al., 1999). Beclin-1 is monoallelically deleted in 40%–70% of human breast, prostate, and ovarian cancers (Liang et al., 1999; Qu et al., 2003; Karantza-Wadsworth et al., 2007; Saito et al., 1993), though biallelic mutations in Beclin-1 are rare in human tumors. Instead, other malignancies often show monoallelic deletions. In high-grade malignancies, such as prostate and ovarian cancers, autophagy tends to be downregulated (Liang et al., 2001; Gao et al., 1995). An initial study indicated that inhibiting Beclin-1 accelerated the progression of premalignant lesions caused by agents like the hepatitis B virus, enhanced the emergence of spontaneous cancers in the lung, liver, and lymphomas, and promoted mammary hyperplasia (Liang et al., 1999). This highlights how dysregulation of Beclin-1 and autophagy genes contributes to the development of human cancers. Subsequent research has linked abnormal autophagy to inflammation, DNA damage, genetic instability, insufficient cell turnover, and the production of reactive oxygen species (ROS), all of which are precursors to tumorigenesis and cancer (Table 2) (Karantza-Wadsworth et al., 2007).

**TABLE 2:** Autophagy with dual function in cancer progression and suppression.

Autophagy action	Highlight	Reference
Pro-death	CircTICRR suppresses autophagy through HuR binding and increasing GLUD1 stability Silencing circTICRR induces autophagy to increase apoptosis	Zhu et al. (2022)
Pro-death	Autophagy can reduce the oncogenic function of YAP in pancreatic tumor	Sun et al. (2021a)
Pro-death	COPZ1 deficiency increases NCOA4 expression to induce autophagy and ferroptosis in glioblastoma	Zhang et al. (2021)
Pro-survival	Platycodin D impairs autophagy through LDLR overexpression to facilitate cell death in glioblastoma	Lee et al. (2022a)
Pro-death	Sendeng-4 stimulates autophagy and apoptosis to reduce the progression of melanoma	Du et al. (2021)
Pro-death	TSPAN9 accelerates autophagy to elevate 5-fluorouracil sensitivity in gastric cancer	Qi et al. (2020)
Pro-survival	The suppression of protective autophagy promotes apoptosis induction by melatonin in the treatment of glioblastoma	Zhou et al. (2019)
Pro-survival	Angelicin stimulates mTOR signaling to inhibit autophagy in cancer therapy	Wang et al. (2019a)
Pro-survival	LncRNA MITA1 mediates protective autophagy in lung cancer in elevating gefitinib resistance	Hu et al. (2021)
Pro-death	TIGAR downregulation by decitabine can promote apoptosis and autophagy in leukemia	Li et al. (2021)

## 5 Chemoresistance regulation by autophagy

Autophagy plays a role in drug resistance in cancer, with chemotherapeutic drugs often limited in their effectiveness due to their induction of protective autophagy, leading to chemoresistance (Hill and Wang, 2020). For instance, cisplatin, commonly used in treating various cancers including ovarian cancer, activates autophagy through the ERK pathway, thereby promoting drug resistance in these cells (Wang and Wu, 2014). Inhibition of autophagy has been shown to sensitize cancer cells to cisplatin (Bao et al., 2015; You et al., 2019), with similar results in lung cancer (Lee et al., 2015). In esophageal cancer, cisplatin-induced autophagy via the class III PI3K pathway enhances treatment efficacy when combined with the autophagy inhibitor 3-Methyladenine (Liu et al., 2011). Similarly, 5-FU, which inhibits DNA synthesis (Park et al., 2013), also induces autophagy leading to chemoresistance (Shuhua et al., 2015). Blocking autophagy has enhanced the effectiveness of 5-FU in colorectal cancer, where ATG genes have been linked to multi-drug resistance (Li et al., 2010). Activation of c-Jun N-terminal kinases (JNK) and phosphorylation of Bcl-2 are key mechanisms in 5-FU-induced autophagy in colon cancer, providing protection to cancer cells (Park et al., 2013). This phenomenon is also observed in gallbladder carcinoma, where inhibiting autophagy with chloroquine enhances the cytotoxic effects of 5-FU (Liang X. et al., 2014). In estrogen receptor-positive breast cancer, suppression of autophagy can resensitize cells to tamoxifen (Samaddar et al., 2008). In prostate cancer, elevated levels of the tumor suppressor candidate gene, nitrogen permease regulator-like 2, increase resistance to Everolimus by enhancing autophagy via the mTOR pathway (Chen et al., 2019). Autophagy also interacts with apoptosis, often protecting cancer cells from drug-induced cell death. In breast cancer, treatment with Epirubicin induces autophagy in MCF-7 cells, shielding them from apoptosis. However, inhibition of autophagy can resensitize these drug-resistant cells to therapy (Sun et al., 2011). In osteosarcoma, common chemotherapeutics induce overexpression of HSP90AA1, regulating autophagy through the PI3K/Akt/mTOR pathway and apoptosis through JNK/p38, highlighting the intricate interactions of these pathways in drug resistance (Xiao et al., 2018). A comprehensive understanding of these mechanisms is vital for developing new treatments. Novel strategies are emerging that target drug resistance by inhibiting autophagy, enhancing the efficacy of chemotherapy (An et al., 2015; O'Donovan et al., 2011; Zhang et al., 2016; Zhang et al., 2010; Fan et al., 2010; Ahn and Lee, 2011; Carew et al., 2007; Ge et al., 2014). Combining anti-cancer drugs with autophagy inhibitors, such as using cisplatin with autophagy suppression, has increased cytotoxicity in cells (Su et al., 2017; Claerhout et al., 2010). Similarly, pairing 5-FU with the autophagy inhibitor hydroxychloroquine has shown increased effects in colon cancer (Sasaki et al., 2010).

Autophagy is thought to play a crucial role in both the development of cancers and their treatment (Pu et al., 2022). Although many patients experience significant benefits from chemotherapy, acquired drug resistance has become a major obstacle to successful treatment. Numerous studies have demonstrated that a variety of chemotherapeutic agents can induce autophagy (Condello et al., 2020; Ashrafizadeh et al., 2020b), which is linked to increased resistance to chemotherapy. Chemotherapy typically triggers apoptosis in cancer cells, but these cells often initiate autophagy as a defense mechanism to avoid apoptosis, thereby reducing the efficacy of the treatment. Liu et al. (2013) used MTT and Hoechst 33342 staining, along with flow cytometry, to detect apoptosis in A549 lung cancer cells post-chemotherapy. They also employed the autophagy inhibitor 3-methyladenine (3-MA) to explore the relationship between autophagy and apoptosis. Their findings indicated that drugs like cisplatin (DDP) and paclitaxel can induce both autophagy and apoptosis in A549 cells. Additionally, studies have revealed that autophagy can render salivary gland adenoid cystic carcinoma cells resistant to DDP, often leading to chemotherapy failure (Tan et al., 2020). Using transmission electron microscopy, the autophagy marker LC3 can be identified, and the presence of minimal levels of p62 also suggests autophagy triggered by DDP. Moreover, downregulating Beclin-1 using 3-MA or RNA interference has been shown to increase apoptosis induced by DDP. As a result, the activation of protective autophagy by chemotherapy contributes to an increase in chemotherapeutic resistance in tumor cells.

## 6 SIRT1: Cellular functions and oncological importance

### 6.1 Structure and cellular functions

**FIGURE 3:**
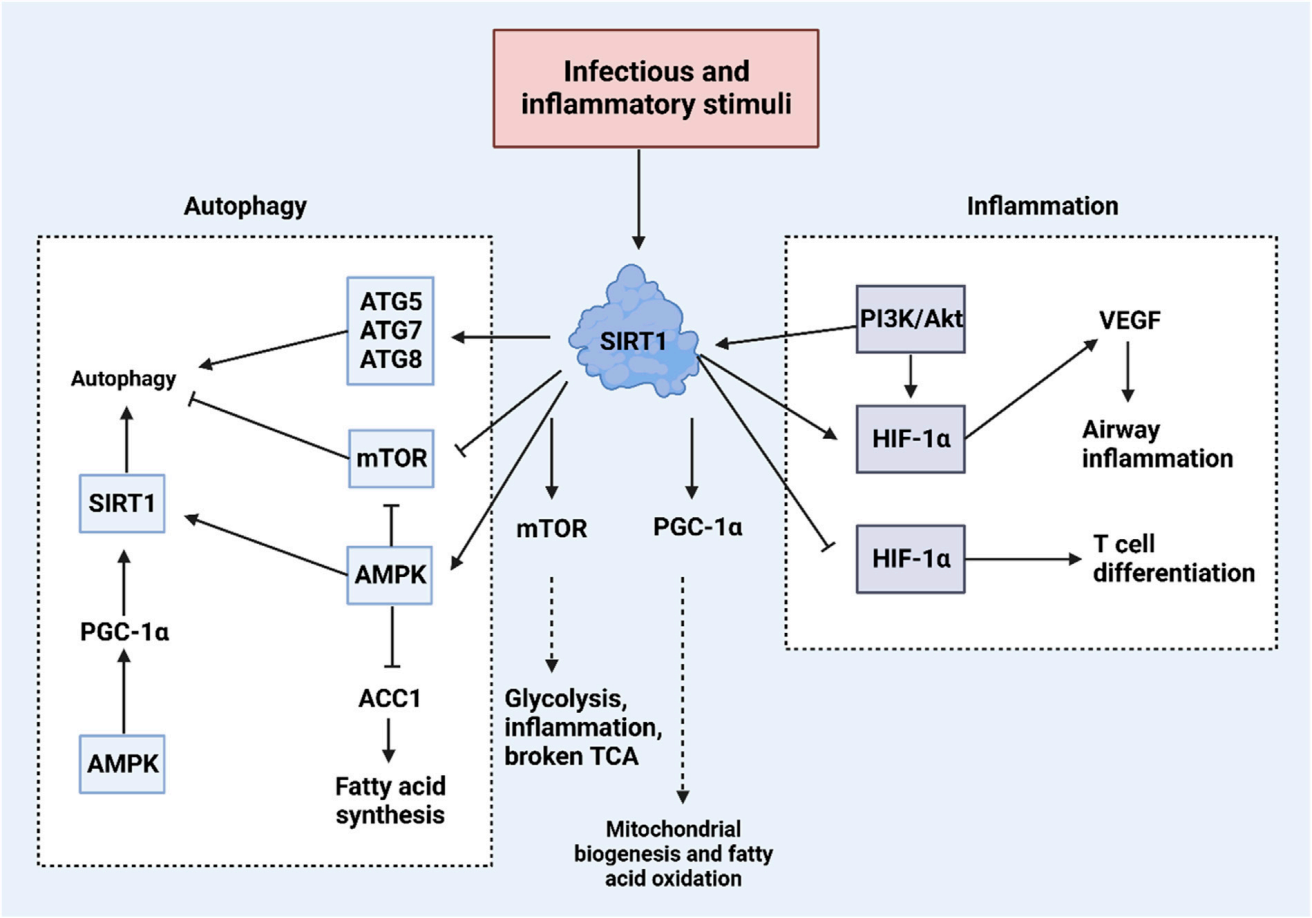
An overview of biological and cellular functions of SIRT1. Exposure to infectious and inflammatory stimuli can lead to an increase in SIRT1 expression, which plays a crucial role in regulating both inflammation and autophagy within cells. SIRT1 enhances autophagy by increasing the levels of ATG proteins such as ATG5, ATG7, and ATG8. Additionally, SIRT1 activates AMPK and suppresses mTOR, further promoting autophagy. In terms of regulating inflammation, SIRT1 interacts with the PI3K/Akt pathway and HIF-1α, illustrating its comprehensive role in cellular response mechanisms. (Kim et al., 2022).

Sirtuins are characterized by a conserved catalytic domain, NAD + binding domains, and variable NH2- and COOH-terminal sections (Jiao and Gong, 2020; Frye, 1999; Yamamoto et al., 2007). These proteins differ in their functions, catalytic activities, and cellular localizations, influenced by their distinct amino acid sequences. Human sirtuins are classified into four categories: Class I, closely related to yeast Sir2, includes SIRT1, SIRT2, and SIRT3; Class II consists of SIRT4; Class III is represented by SIRT5; and Class IV includes both SIRT6 and SIRT7 (Frye, 2000). SIRT1, which is composed of 747 amino acids, features the longest terminal extensions, including a conserved catalytic core (244–512 residues), a COOH-terminal region (1–180 residues), and an NH2-terminal region (513–747 residues) (Kumar and Chauhan, 2016). The nuclear localization signal (KRKKRK) within the 41–46th residues of SIRT1 explains its presence in the nucleus (Frye, 1999). However, SIRT1 is also found in the cytoplasm in some cell types, indicating dual localization (Jin et al., 2007; Moynihan et al., 2005; Stünkel et al., 2007). SIRT1’s ability to shuttle between the nucleus and cytoplasm (Yanagisawa et al., 2018) is regulated by nuclear import and export sequences within its NH2-terminal region (Tanno et al., 2007). Other sirtuins have distinct subcellular locations: SIRT2 typically resides in the cytoplasm, though it can shuttle to the nucleus (North et al., 2003; Inoue et al., 2007); SIRT3, SIRT4, and SIRT5 are primarily mitochondrial, with SIRT3 being shown to move to the mitochondria from the nucleus post UV exposure or etoposide treatment (Scher et al., 2007). SIRT6 and SIRT7, like SIRT1, are located in the nucleus, with SIRT7 localized specifically to the nucleolus and SIRT6 associated with chromatin (Michishita et al., 2005). SIRT1 plays a significant role in regulating various biological and cellular processes, such as aging, metabolism, and inflammation (Chen et al., 2021). Figure 3 illustrates the functions of SIRT1 in these biological events.

### 6.2 Role in cancer

Despite the cellular functions of SIRT1, increasing evidence has underscored its role as a potential regulator of tumorigenesis. SIRT1 interacts with various signaling networks to influence the carcinogenesis process. It is upregulated in colorectal cancer and downregulates p53 expression through deacetylation, reducing miR-101 levels, while enhancing KPNA3 expression to promote metastasis and drug resistance (Wang XW. et al., 2023). Additionally, cytoplasmic SIRT1 may contribute to the formation and survival of polypoid giant tumor cells, leading to paclitaxel resistance in ovarian tumors (Xu H. et al., 2023). Conditions such as glucose deprivation and oxidative stress can trigger SIRT1 upregulation, which mediates β-catenin deacetylation, facilitating its transfer from the nucleus to the cytoplasm to decrease glycolysis and enhance fatty acid oxidation (Wei et al., 2023). Importantly, USP14 can increase the stability of SIRT1 by preventing its deubiquitination, promoting fatty acid oxidation in macrophages, which leads to M2 polarization and tumorigenesis (He et al., 2023). In terms of SIRT1’s oncogenic role, inhibiting it can disrupt tumorigenesis; for instance, LITAF increases FOXO1 levels, leading to SIRT1 downregulation, which diminishes the stemness and malignant phenotype of tumor cells (Guan et al., 2023). SIRT1 also regulates fatty acid oxidation in tumor cells. NSD2 boosts SIRT1 expression through interaction with AROS, enhancing fatty acid oxidation and reducing responsiveness to radiotherapy (Luo H. et al., 2023). Propofol’s potential as an anti-cancer agent in reducing tumor metastasis is partly attributed to the downregulation of SIRT1 (Wang R. et al., 2023). The transfer of SIRT1 via extracellular vesicles can activate the CD24/Siglec-10 axis, increasing apoptosis in CD8^+^ T cells and accelerating carcinogenesis (Zheng Q. et al., 2024). Moreover, SIRT1 regulates cell death mechanisms in cancers, such as inhibiting ferroptosis via p53 downregulation, thereby enhancing the survival of gastric tumor cells (Zhao H. et al., 2023). The following sections will delve deeper into the role of SIRT1 in autophagy regulation and associated molecular pathways (Table 3).

**TABLE 3:** Summarizing the underlying mechanisms involved in SIRT1-mediated cancer regulation.

Targets	Highlights	References
SIRT1/WEE1	SIRT1 mediates WEE1 deacetylation to increase sensitivity to WEE1 suppression	Zhu et al. (2023a)
NCAPD2/PARP-1/SIRT1	NCAPD2 disrupts autophagy mechanism through controlling PARP-1/SIRT1 axis	Jing et al. (2021)
SIRT1	The overexpression of SIRT1 in liver cancer enhances energy homeostasis and regulates antioxidant response	Varghese et al. (2023)
SIRT1/p53/miR-101/KPNA3	SIRT1 induces drug resistance in colorectal tumor through p53 downregulation to reduce miR-101 levels in upregulating KPNA3	Wang et al. (2023d)
SIRT1	SIRT1 enhances tumorigenesis in colorectal cancer via enhancing glucolipid metabolic conversion	Wei et al. (2023)
SIRT1	Cytoplasmic SIRT1 promotes the formation and viability of polyploidy giant cancer cells to mediate paclitaxel resistance in ovarian cancer	Xu et al. (2023b)
LITAF/FOXO1/SIRT1	LITAF inhibits SIRT1 by FOXO1 to reduce proliferation and metastasis in colorectal tumor	Guan et al. (2023)
SIRT1/STAT3/MMP-13	SIRT3 disrupts the growth and invasion of gastric cancer through STAT3 inhibition to induce MMP-13 expression	Zhang et al. (2019)
NSD2/AROS/SIRT1	NSD2 facilitates AROS methylation to upregulate SIRT1	Li et al. (2023a)

When compared to the non-cancerous tissues that were next to EC tissues, ENST00000534735 in EC tissues was dramatically downregulated (Shan et al., 2024). In addition to facilitating apoptosis and pyroptosis, the ectopic expression of ENST00000534735 significantly stopped the capacity of lung cancer cells to proliferate and migrate. The elevation of OSBPL3 through the APMK/SIRT1/NF-κB pathway was able to counteract the tumor-suppressing effects of ENST00000534735 overexpression. This was accomplished by knocking down ENST00000534735, which resulted in an increase in OSBPL3 expression. An excessive amount of ENST00000534735 expression was shown to inhibit the development of EC in the *in vivo* tumorigenic experiments that were carried out on nude mice. Another study identifies SIRT1 as a target of ISGylation, a post-translational modification by ISG15, which enhances SIRT1’s deacetylase activity by disrupting its interaction with the inhibitor DBC1 (Kang et al., 2024). SIRT1 ISGylation promotes lung cancer progression and reduces the sensitivity of lung cancer cells to DNA damage-based therapies. Elevated ISG15 and SIRT1 levels in lung cancer tissues correlate with poor patient prognosis, suggesting that these biomarkers could aid in patient stratification and outcome evaluation. SIRT1 downregulation in oral cancer cells leads to mitochondrial hyperfusion and drug resistance, while SIRT1 overexpression or activation by gallic acid reverses this effect, promoting apoptosis and restoring cisplatin sensitivity (Patra et al., 2023a). SPC-180002, a novel dual inhibitor of SIRT1/3, disrupts redox homeostasis and mitochondrial function, leading to cell cycle arrest and strong inhibition of cancer cell growth (Cho et al., 2023). MiR-653–3p promotes genomic instability, proliferation, migration, and chemoresistance in colorectal cancer cells by inhibiting SIRT1 and activating the TWIST1 signaling pathway (Wang H. et al., 2023). Doxorubicin-induced SIRT1 promotes redox imbalance and chemoresistance in breast cancer by enhancing cell proliferation, angiogenesis, and metastasis through NRF2 activation and increased glutathione levels (Sahoo et al., 2024). SIRT1 deacetylates and enhances KRASMut activity in lung cancer, and inhibiting SIRT1 or activating p300, which acetylates KRASMut, sensitizes tumors to cisplatin and erlotinib, offering a potential combination therapy for KRASMut lung cancer (Shin et al., 2023). Resveratrol inhibits neutrophil extracellular trap formation by targeting SIRT1, thereby reducing breast cancer metastasis and promoting CD8^+^ T cell infiltration in a murine model (Yu W. et al., 2023). Therefore, increasing evidences highlight the function of SIRT1 in the regulation of cancer progression and interaction with various molecular pathways (Wang XW. et al., 2023; Xu H. et al., 2023; Li X. et al., 2023; Zhang X. et al., 2023; Liu S. et al., 2024).

## 7 General discussion of SIRT1 in autophagy regulation in cancer

The process of mitotic chromosomal condensation is largely dependent on the presence of condensin (Hirano, 2002). Condensin I and condensin II are the names given to the two distinct forms of condensin complexes that may be found in a wide variety of eukaryotic cells (Hirano et al., 1997). The conventional condensin complex is composed of three distinct non-SMC subunits in addition to the same pair of core subunits that are referred to as structural maintenance of chromosomes (SMC) family proteins (Kimura and Hirano, 2000). Within human cells, the non-SMC subunits of condensin I are denoted by the letters NCAPD2, NCAPG, and NCAPH. On the other hand, the comparable subunits in the condensin II complex are denoted by the letters NCAPD3, NCAPG2, and NCAPH2 (Hirano et al., 1997). Condensin I has three non-SMC subunits, and one of them is called NCAPD2. This component may be found on chromosome 12p13.3. Previous research on NCAPD2 has mostly concentrated on its role in mitotic chromosomal condensation and segregation. This is because NCAPD2 is an essential component of the cell cycle. In addition, a number of studies have demonstrated that NCAPD2 is linked to a number of neurodevelopmental diseases, including Alzheimer’s disease, autism, Parkinson’s disease, and others, which suggests that it may have a function in the development of the central nervous system (Lee et al., 2008; Li et al., 2009; Sanders et al., 2012; Zhang et al., 2014). The abnormal expression of NCAPD2 in triple-negative breast cancer has the potential to function as an independent prognostic factor (Zhang et al., 2020). Through its involvement in the Ca2+/CAMKK/AMPK/mTORC1 pathway and the PARP-1/SIRT1 axis, NCAPD2 is able to suppress autophagy and impede autophagic flux. NCAPD2 is a tumor promoter that may be found in both *in vitro* and *in vivo* settings. In an AOM/DSS-induced mouse model, suppression of the development of colorectal cancer by NCAPD2 deletion is seen (Jing et al., 2021). 4-dmH targets tNOX and SIRT1, inhibiting their activity and inducing apoptosis (Islam et al., 2024a). SIRT1 in EML4-ALK G1202R and EML4-ALK L1196M mutant drug-resistant cells was downregulated compared with EML4-ALK NSCLC cells (Yang et al., 2024). The high expression of SIRT1 was related to the longer survival time of patients with lung cancer. Activation of SIRT1 induced autophagy and suppressed the invasion and migration of mutant cells. Further experiments indicated that the activation of SIRT1 inhibited the phosphorylation level of mTOR and S6K by upregulating the expression of AMPK, thus activating autophagy. SIRT1 can significantly enhanced the sensitivity of mutant cells to crizotinib, improved its ability to promote apoptosis of mutant cells, and inhibited cell proliferation.

A number of transcription factors, including p53, E2F1, FOXO, NF-θβ, and c-Myc, have been identified as targets for SIRT1 (Mao et al., 2014). These interactions are responsible for the formation of cancer and the spread of disease to other parts of the body in a variety of malignancies (Ayob and Ramasamy, 2018; Wong et al., 2021; Ong and Ramasamy, 2018). Overexpression of SIRT1 in HCC has the potential to contribute to the survival and proliferation of tumor cells (Chen et al., 2011; Jang KY. et al., 2012; Molla et al., 2020), as well as to the promotion of metastasis (Hao et al., 2014). SIRT1 is mostly found in the nucleus, where it plays a function in the development of tumors. However, it has been suggested that cytoplasmic sirtuin 1 may play a role in the suppression of tumors in HCC (Farcas et al., 2019; Song et al., 2014). SIRT1 is also known to influence chemoresistance in a variety of malignancies, including ovarian, breast, and gastric cancers (An et al., 2020; Mvunta et al., 2017; Wang et al., 2019b). However, the involvement of SIRT1 in the chemoresistance of HCC is not well understood. A study investigates the role of SIRT1 in sorafenib-resistant HCC, revealing that increased SIRT1 levels promote autophagy and activate NF-ĸβ signaling in resistant cells (Chan et al., 2024). Silencing SIRT1 downregulates autophagy and restores NF-ĸβ activity by failing to deacetylate key proteins, suggesting that the SIRT1/autophagy/NF-ĸβ axis plays a crucial role in HCC progression and resistance, with potential implications for therapeutic strategies.

There was a significant amount of RACGAP1 found in the cells of stomach cancer. Gastric cancer cell proliferation, migration, and invasion were all enhanced when RACGAP1 was overexpressed (Yan et al., 2024). In addition, the inhibition of RACGAP1 led to the induction of autophagy and death in cells. In addition, the expression of SIRT1 and Mfn2 was also inhibited by RACGAP1. In the tissues of EC tumors, FIRRE and SIRT1 were found to be elevated, whereas miR-199b-5p was shown to be downregulated. By sponging miR-199b-5p and suppressing autophagy, FIRRE knockdown was able to improve the susceptibility of EC cells to radiation doses (Cai et al., 2024). The microRNA known as miR-199b-5p was able to act as a negative regulator of SIRT1. In the absence of this information, SIRT1 has the potential to deacetylate BECN1 protein and take part in FIRRE-mediated autophagy. The activation of FIRRE resulted in an enhancement in the sensitivity of EC radiation *in vivo*. By inhibiting autophagy and proliferation, as well as inducing apoptosis in HCT116 and HT29 cells, ZMIZ1 knockdown was found to have a substantial therapeutic effect (Huang et al., 2024). Both the mRNA level of SIRT1 and the protein level of the SIRT1-specific substrate, acetylated FOXO3a, were considerably reduced as a result of ZMIZ1 knockdown. However, the mRNA level of SIRT1 was not changed by the knockdown. The relationship between SIRT1 and ZMIZ1 in HCT116 and HT29 cells was brought to light by immunoprecipitation tests. There was an increase in the intracellular ubiquitination of SIRT1 due to ZMIZ1. The effects of ZMIZ knockdown on proliferation, autophagy, and apoptosis in HCT116 and HT29 cells were reduced by targeting SIRT1 by knockdown or pharmacological inhibition. The drug-resistant oesophageal cancer cells exhibit increased autophagy and SIRT1 expression, both of which are linked to enhanced cell migration and the EMT (Zhang et al., 2024b). Inhibiting autophagy or SIRT1 reduced these processes. Additionally, a SIRT1 inhibitor effectively suppressed tumor growth in a mouse xenograft model without significant toxicity, suggesting that SIRT1 plays a key role in autophagy-driven drug resistance in oesophageal cancer. The adipose triglyceride lipase (ATGL) is highly expressed in CRC and is associated with poor prognosis (Su BC. et al., 2023). ATGL promotes CRC cell proliferation by inhibiting the mTOR signaling pathway and activating autophagy. Additionally, ATGL regulates autophagy by increasing SIRT1 expression. These findings suggest that ATGL contributes to CRC growth through the upregulation of autophagy and SIRT1. The electro-acupuncture (EA) can alleviate CRC in mice by reducing inflammation and promoting autophagy through the SIRT1/miR-215/Atg14 axis (Li J. et al., 2023). EA treatment decreased tumor numbers, inflammation, and DAI scores, while increasing body weight and SIRT1 expression. SIRT1 overexpression was shown to suppress miR-215 and enhance Atg14 expression, suggesting that EA exerts its anti-CRC effects by regulating this molecular pathway. The ubiquitin-conjugating enzyme E2C (UBE2C) promotes the malignant progression of endometrial cancer by inhibiting autophagy (Zhao R. et al., 2023). UBE2C suppresses autophagy by inducing ubiquitination and degradation of SIRT1, leading to reduced expression of autophagy-related genes. Knockdown of UBE2C in cancer cells enhanced autophagy and increased apoptosis, while overexpression of UBE2C promoted tumor growth in a mouse model. However, rapamycin, an autophagy activator, reversed the tumor growth and apoptosis inhibition caused by UBE2C overexpression. SIRT1 regulates mitotic catastrophe (MC) through autophagy and BubR1 signaling. Degradation of SIRT1 increased MC, while overexpression of SIRT1 reduced MC by decreasing apoptotic and multinuclear cells and promoting autophagy. Additionally, SIRT1 was shown to bind to the promoter of BubR1, a key component of the spindle assembly checkpoint, increasing its expression and reducing MC (Zhao et al., 2022).

## 8 SIRT1/AMPK axis in autophagy regulation

AMPK, a crucial metabolic regulator, restores depleted ATP levels and maintains energy balance, especially when cells are stressed. Targeting AMPK has shown promise in treating metabolic syndrome and type 2 diabetes (Steinberg and Kemp, 2009; Yuan et al., 2023). AMPK enhances metabolic processes by inhibiting glucose production in the liver, improving insulin sensitivity, reducing fatty acid synthesis and esterification, increasing glucose uptake in muscles, and reducing proinflammatory changes (Ruderman and Prentki, 2004). Small molecules such as cellular AMP allosterically activate AMPK by binding to the CBS1 domain, while AMP or ADP binding to CBS3 alters AMPK’s phosphorylation status (Xiao et al., 2011). These interactions trigger structural changes in the AMPK complex, enabling phosphorylation at the Thr-172 site on the AMPKα subunit (Hawley et al., 1996; STEIN et al., 2000), and are further enhanced by various upstream kinases that also phosphorylate the Thr-172 site, fully activating AMPK (Liu et al., 2014). AMPK acts as a regulator of autophagy in various cancers, with growing evidence suggesting that SIRT1 serves as an upstream mediator of AMPK in this role. Quercetin, a natural compound, induces apoptosis and toxic autophagy in lung cancer, where increased SIRT1 levels upregulate AMPK, leading to autophagy-mediated apoptosis (Guo et al., 2021). Similarly, ghrelin enhances SIRT1 expression to activate AMPK and induce autophagy, although this SIRT1/AMPK-mediated autophagy does not significantly trigger apoptosis (Heshmati et al., 2020). The SIRT1/AMPK axis has been studied across different tumor types, influencing tumorigenesis progression. For example, diallyl trisulfide induces pro-death autophagy in hepatocellular carcinoma through the AMPK/SIRT1 axis (Sun et al., 2022). Additionally, since mTOR is downstream of AMPK, SIRT1’s regulation of AMPK impacts mTOR, a key autophagy regulator (Ye et al., 2017). Calycosin activates the SIRT1/AMPK axis to inhibit the Akt/mTOR pathway, stimulating autophagy-mediated apoptosis in cancer cells (El-Kott et al., 2019). Nitrosative stress can also induce autophagy in breast cancer by upregulating SIRT1 and its interaction with AMPK (Chakraborty et al., 2019). Thus, SIRT1 is integral in regulating AMPK and downstream targets, influencing autophagy in human cancers.

## 9 SIRT1/mTOR axis in autophagy regulation

**FIGURE 4:**
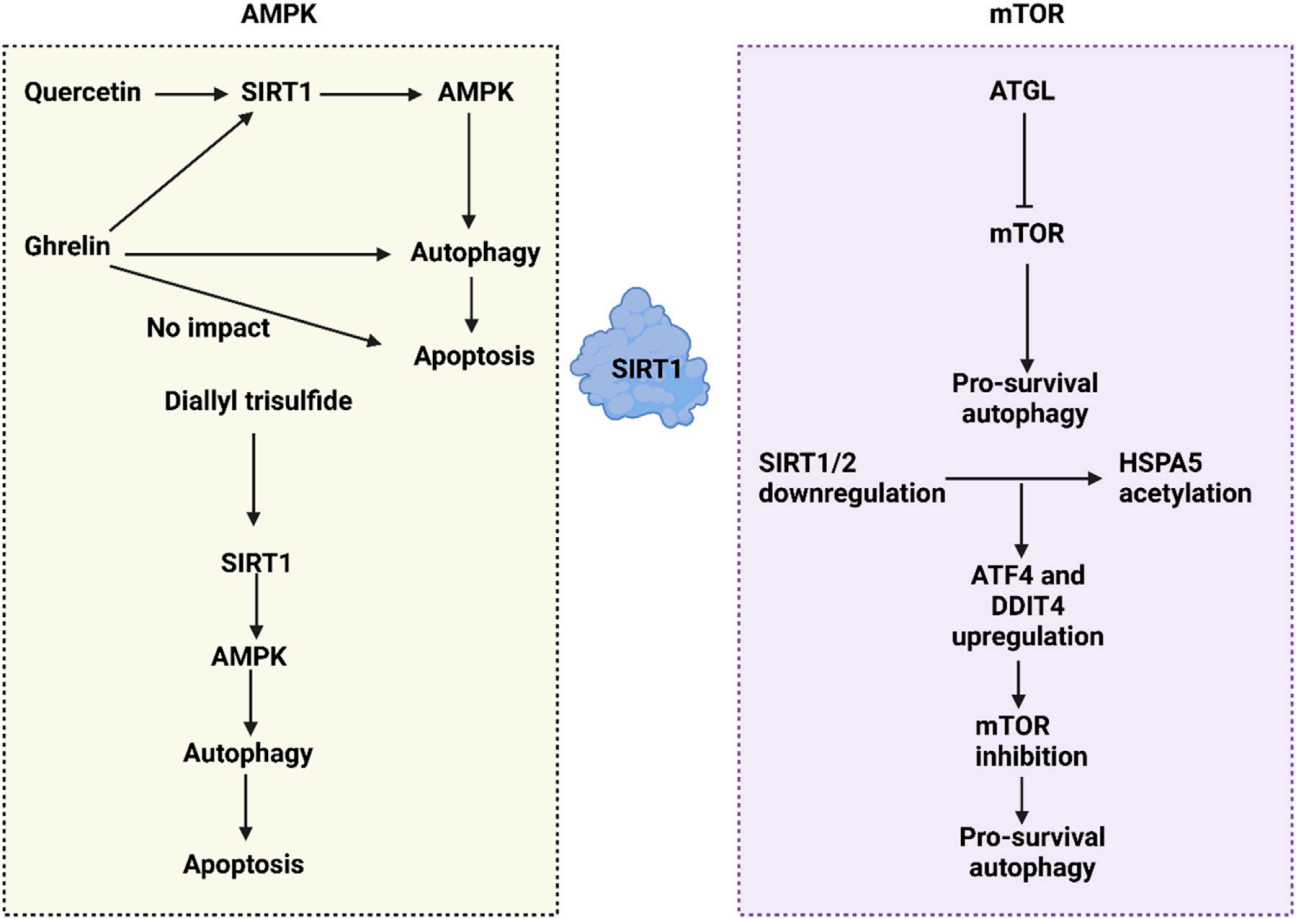
The SIRT1-mediated autophagy regulation in cancer through affecting mTOR and AMPK pathways. The interaction between SIRT1 and AMPK clearly illustrates that SIRT1 upregulates AMPK to promote autophagy. Various compounds influence the SIRT1/AMPK axis; for example, quercetin activates the SIRT1/AMPK/autophagy pathway to stimulate apoptosis. Ghrelin also activates the SIRT1/AMPK/autophagy axis, though it does not lead to cell death. Additionally, the downregulation of SIRT1/2 enhances the acetylation of HSPA5, which in turn increases ATF4 and DDIT4 levels, leading to the downregulation of mTOR and facilitating pro-survival autophagy.

mTOR, a highly conserved serine/threonine kinase, orchestrates cellular metabolism, proliferation, and apoptosis (Xie et al., 2023). It forms two distinct complexes: mTOR complex 1 (mTORC1) and mTOR complex 2 (mTORC2), with mTORC1 being more sensitive to rapamycin and containing the regulatory-associated protein of mTOR (RAPTOR) (Ben-Sahra and Manning, 2017). mTOR responds to three main types of upstream signals: immune activation, environmental stress, and nutrient availability (Chi, 2012). These signals can either upregulate or downregulate mTOR, influencing cell growth, division, and survival, as well as regulating protein synthesis and catabolism. Downstream of mTOR, translational regulation is mediated by factors such as the eIF4E binding protein 1 (4E-BP1) and p70S6 Kinase (S6 Kinase), illustrating another facet of mTOR signaling (Zou et al., 2020; Tan and Miyamoto, 2016; Kennedy and Lamming, 2016). SIRT1 interacts with mTOR to regulate autophagy in human cancers. For instance, ATGL, identified as an oncogenic factor in colorectal tumors, promotes proliferation and correlates with poor prognosis by downregulating mTOR, thus facilitating pro-survival autophagy (Su BC. et al., 2023). SIRT1 regulators have emerged as autophagy modulators in cancer. MHY2245, an inhibitor of SIRT1, suppresses the PKM2/mTOR axis, stimulating autophagy and accelerating apoptosis, which leads to growth reduction in ovarian tumors (Yousafzai et al., 2021). The downregulation of SIRT1/2 can induce protective autophagy in lung cancer by increasing the acetylation of HSPA5, which in turn elevates ATF4 and DDIT4 levels, suppressing mTOR and promoting pro-survival autophagy (Mu et al., 2019). Thus, both AMPK and mTOR play significant roles in the regulation of autophagy in human cancers (Figure 4).

## 10 SIRT1-mediated autophagy regulation in cancer drug resistance

A major challenge in oncology is the issue of drug resistance, a problem that shares similarities with antimicrobial therapy in terms of rapidly adapting threats, primarily originating from within, such as cancerous cells, and to a lesser extent from external sources like bacteria. Early chemotherapeutics such as nitrogen mustard and aminopterin were initially effective, putting many tumors into remission. However, similar to antimicrobial chemotherapy, they often led to drug resistance and disease relapse. Drawing on strategies from antimicrobial therapy, oncology first attempted to overcome resistance through polychemotherapy, which involves administering a sequence of drugs, each with a different mechanism of action. This approach has been empirically successful in treating certain diseases like some types of lymphoma, breast cancer, and testicular cancer. Consequently, combination chemotherapy became the foundation of systemic cancer treatment, frequently used alongside surgery and tailored radiation therapy. Over time, these combinations grew more complex, and dose intensity strategies were introduced to enhance antitumor efficacy. This involved reducing intervals between chemotherapy cycles or increasing drug dosages, supported by myeloid and other growth factors to manage drug-induced myelotoxicity and sustain ongoing treatment. Despite nearly 5 decades of success, by the early 21st century, it became evident that surgery, radiation, and combination chemotherapy were not curative for many types of tumors (Vasan et al., 2019; Goodman et al., 1946; Farber and Diamond, 1948; Crofton, 1959; DeVita et al., 1980; Bonadonna et al., 1976; Bosl et al., 1986; Hryniuk and Bush, 1984; Citron et al., 2003; Sternberg et al., 2001).

Chemotherapy drugs induce mitochondrial dysfunction to trigger apoptosis in tumor cells. Conversely, CDK9 inhibitors enhance the stability and dephosphorylation of SIRT1. Subsequently, elevated SIRT1 levels lead to the degradation of FOXO3, which in turn suppresses BNIP3-induced stability of PINK1. Additionally, CDK9 inhibitors can inhibit the SIRT1/FOXO9/BNIP3 axis and the PINK1/PRKN pathway, thereby suppressing mitophagy. This inhibition of mitophagy by CDK9 inhibitors contributes to increased mitochondrial dysfunction, ultimately promoting apoptosis in hepatocellular carcinoma (Yao et al., 2022). Despite evidence suggesting that increased stability and upregulation of SIRT1 can suppress mitophagy and enhance apoptosis, some findings overexpressed SIRT1 can facilitate drug resistance in tumor cells. Specifically, SIRT1 can mediate the deacetylation of Beclin-1, which activates protective autophagy and promotes resistance to cisplatin in bladder cancer (Sun et al., 2023).

Autophagy induction during tumorigenesis serves to supply cancer cells with the necessary components for growth by degrading organelles and proteins (White et al., 2015). Modifying autophagy levels has emerged as a promising strategy in cancer treatment (Li et al., 2017). Lipophagy, a selective form of autophagy that degrades lipids, plays a role in modulating lipid metabolism and maintaining intracellular lipid homeostasis (Zhang et al., 2018). Various genes, enzymes, transcription regulators, and other molecules regulate lipophagy (Maan et al., 2018; Madrigal-Matute and Cuervo, 2016). Additionally, *de novo* lipogenesis is linked to the development of drug resistance in cancer (Zhang et al., 2018; Maan et al., 2018; Beloribi-Djefaflia et al., 2016). For instance, low expression of miR-425 can elevate SIRT1 levels, thereby stimulating pro-survival lipophagy and enhancing resistance to sorafenib in liver cancer (Sun G. et al., 2021). Conversely, the independent regulation of SIRT1 and autophagy can also influence drug resistance. Jaridon 6, for example, inhibits the PI3K/Akt/mTOR axis to induce autophagy and reduces SIRT1 expression, weakening drug resistance in gastric tumors (Fu et al., 2021). A notable aspect of SIRT1-mediated autophagy is its role in various human cancers. The lncRNA H19, which has oncogenic properties in colorectal cancer, mediates 5-fluorouracil resistance by sponging miR-194–5p to elevate SIRT1 expression, thus promoting autophagy-induced resistance to 5-fluorouracil in colorectal tumors (Wang et al., 2018). Thus, the interaction between SIRT1 and autophagy plays a critical role in determining the responsiveness of tumor cells to chemotherapy.

## 11 SIRT1-mediated autophagy and apoptosis crosstalk

### 11.1 Basics of apoptosis

During the initiation and intermediate stages of apoptosis, a variety of metabolic activities occur alongside significant morphological changes. These changes include cytoplasmic filament aggregation, nuclear membrane shrinkage, cell fragmentation, the formation of apoptotic bodies, and plasma membrane blebbing (Elmore, 2007; Power et al., 2002). These changes are mainly observed in the nucleus, cell membrane, cytoplasm, and mitochondria, and can be detected through microscopic, light, and fluorescence microscopy methods (Elmore, 2007; Savill and Fadok, 2000). Apoptosis is triggered by environmental signals originating from two primary sources: external signals from other cells and signals from physical contact with adjacent cells. At the onset of apoptosis, cells begin to lose contact with neighboring cells and tightly pack their internal components without releasing them outside, thus preventing inflammation and contamination in the surrounding environment (Rosenblatt et al., 2001; Ferri and Kroemer, 2001). Surrounding cells recognize these apoptotic cells and facilitate their internalization and degradation without triggering an inflammatory response. Apoptosis proceeds via two major pathways: intrinsic and extrinsic. The extrinsic pathway is activated by the interaction of death receptors with their ligands, leading to the activation of caspase 8. This activation can directly induce cell death or further activate caspase 3 or Bid, a process that can be inhibited by cellular FLICE-like inhibitory proteins (cFLIP) (Kiraz et al., 2016). The intrinsic pathway, on the other hand, is initiated by genomic damage and proceeds via the mitochondrial pathway. This involves the activation of Bax, a pro-apoptotic member of the Bcl-2 family. At the mitochondrial membrane, anti-apoptotic proteins such as Bcl-2 and Bcl-XL inhibit Bax’s activity. The release of cytochrome c from mitochondria leads to the formation of apoptosomes, complexes involving cytochrome c, APAF-1, and procaspase 9. The assembly of these complexes triggers the caspase activation cascade, converting procaspase-3 to active caspase 3. Bid, another pro-apoptotic Bcl-2 family member, facilitates communication between the intrinsic and extrinsic pathways. Caspase 8 cleavage of Bid enhances the release of mitochondrial cytochrome C, further driving the apoptotic process (Kiraz et al., 2016).

### 11.2 SIRT1-mediated autophagy and apoptosis crosstalk

The interactions between autophagy and apoptosis play a crucial role in human cancers, with SIRT1 acting as a key mediator. This section discusses the relationship between SIRT1-induced autophagy and apoptosis. SIRT1 upregulation is essential for initiating autophagy. UBE2C promotes the ubiquitination of SIRT1, leading to its degradation and decreased stability, which in turn reduces H4K16 deacetylation, suppressing autophagy at an epigenetic level. In endometrial cancer, autophagy is critical for inducing apoptosis, hence UBE2C’s regulation of autophagy affects the autophagy-apoptosis interplay (Zhao R. et al., 2023). In some cases, autophagy can inhibit apoptosis in human cancers. For instance, SIRT1 translocation from the nucleus to the cytoplasm increases Beclin-1 expression, thereby promoting autophagy. This protective autophagy then inhibits the release of cytochrome C from mitochondria, suppressing the caspase-3/PARP pathway and preventing apoptosis in bladder cancer (Sun et al., 2023). Additionally, the response of autophagy to cellular stress is pivotal, as evidenced by increased SIRT1 and FoxO1 levels under glucose deprivation in gastric cancer, which boosts Rab7 expression and autophagy, supporting tumor cell survival. Conversely, inhibiting autophagy can enhance apoptosis, underscoring the supportive role of autophagy in this context (Zhu M. et al., 2023). In colorectal cancer, SIRT1 typically stimulates autophagy to inhibit apoptosis. However, using catalpol, a natural product with anticancer and epigenetic properties, leads to miR-34a upregulation, which suppresses the SIRT1/autophagy axis and triggers apoptosis in colorectal tumor cells (Qiao et al., 2020). Although SIRT1 is primarily seen as an upstream autophagy mediator in cancers, autophagy can also influence SIRT1, impacting tumorigenesis regulation. For example, autophagy-induced SIRT1 degradation can enhance radiotherapy-mediated apoptosis in prostate cancer, showing its potential to reduce radio-resistance (Wang et al., 2022). Epigenetic modifications and miRNA dysregulation in tumor cells also affect cancer progression and treatment responses (Ashrafizadeh et al., 2021). In lung cancer, miR-124 and miR-142 downregulation of SIRT1 suppresses supportive autophagy, enhancing cisplatin sensitivity and promoting apoptosis (Song et al., 2019). Furthermore, the anticancer compound elaiophylin decreases SIRT1 and its downstream target Nrf2, inhibiting mitophagy and accelerating apoptosis in lung tumors (Ji et al., 2022). Thus, the interplay between SIRT1-mediated autophagy and apoptosis is integral to the regulation of carcinogenesis (Figure 4).

## 12 SIRT1-mediated autophagy and ferroptosis crosstalk: New perspectives

Ferroptosis, an iron-dependent form of regulated cell death characterized by the accumulation of lipid peroxides on cellular membranes, was first identified in a proteomics study by the Stockwell laboratory and colleagues in 2012 (Dixon et al., 2012; Lei et al., 2022). This process is distinct from apoptosis and other forms of cell death in several ways, including its unique mechanisms and morphological characteristics. Cells undergoing ferroptosis do not exhibit chromatin condensation or form apoptotic bodies; instead, they typically have smaller mitochondria with fewer mitochondrial cristae compared to normal or apoptosis-resistant cells (Dixon et al., 2012; Stockwell et al., 2017). These cells also accumulate harmful lipid peroxides (Jiang et al., 2021), arising from an imbalance between antioxidant activities that prevent ferroptosis and the pro-ferroptotic processes. When the imbalance exceeds the cell’s capacity to cope, leading to a critical overload of lipid peroxides, ferroptosis is triggered (Yang et al., 2014; Bersuker et al., 2019; Doll et al., 2019; Kraft et al., 2020; Soula et al., 2020; Mao et al., 2021; Ingold et al., 2018). Additionally, ferroptosis differs in its molecular mechanisms from other types of cell death, which involve specific executioner proteins like caspase in apoptosis, gasdermin D in pyroptosis, or MLKL in necroptosis. Furthermore, the oxidized phospholipid profiles are distinctive to ferroptosis, setting it apart from other cell death types (Galluzzi et al., 2018; Wiernicki et al., 2020; Kagan et al., 2017).

Recent research has underscored the interplay between autophagy and ferroptosis in various human cancers, illuminating their roles in tumorigenesis regulation. In lung cancer, inducing ferroptosis has been shown to curb tumor growth, with curcumin enhancing this process by promoting toxic autophagy in lung tumor cells (Tang X. et al., 2021). In ovarian cancer, studies have investigated the expression levels of C-MYC and NCOA4 and their relationship with cancer malignancy. Findings indicate a significant correlation, where C-MYC appears to suppress NCOA4 expression by directly interacting with its mRNA, influencing ferroptosis negatively. This interaction reduces NCOA4 levels, decreases ROS production, and inhibits mitophagy, leading to increased proliferation and invasion of ovarian cancer cells. Furthermore, C-MYC is implicated in reducing NCOA4-mediated ferroptosis, enhancing cancer cell invasion and immune evasion (Jin et al., 2022). In head and neck cancer, the induction of ferritinophagy, a specific form of autophagy, is crucial for promoting ferroptosis (Lee J. et al., 2022). Conversely, in cervical cancer, Cdc25A enhances PKM2 dephosphorylation, which upregulates ErB2 expression and inhibits autophagy-induced ferroptosis (Wang et al., 2021). Additionally, in bladder cancer, although GPX4 acts to inhibit ferroptosis, autophagy facilitates the degradation of GPX4, augmenting the efficacy of Fin56 in stimulating ferroptosis (Sun Y. et al., 2021). These findings highlight the complex interactions and crosstalk between autophagy and ferroptosis in cancer regulation. Given the role of SIRT1 as a regulator of autophagy, further exploration into how SIRT1-mediated autophagy might influence ferroptosis is warranted, offering potential new avenues for cancer therapy.

## 13 SIRT1 modulators in cancer

There are various types of sirtuins, with SIRT1 being particularly well-studied for its dual role in cancer progression and inhibition. Researchers have explored pathways to activate or inhibit SIRT1, given its critical regulatory impact on tumor promotion and suppression (Carafa et al., 2019). Recent studies have identified several chemotherapeutic agents that target SIRT1, derived from both synthetic and natural bioactive compounds (Patra et al., 2023b). Among these, the polyphenolic antioxidant resveratrol has been highlighted for its anticancer properties, including antioxidant, immunomodulatory, anti-inflammatory, and pro-apoptotic effects. Resveratrol has shown effectiveness against multiple solid tumors and is known to influence autophagy, suggesting that it might trigger autophagic cell death (ACD) as an alternative cell death mechanism when apoptosis is compromised (Patra et al., 2022; Patra et al., 2021). This activation of SIRT1 by resveratrol could be particularly useful in treating drug-resistant cancer cells and eliminating cancer stem cells (Pervaiz and Holme, 2009). Another agent, gallic acid, known for inhibiting autophagy flux, can also activate SIRT1 and induce ATG cell death (Patra et al., 2020a; Patra et al., 2020b; Chang et al., 2021). Additionally, synthetic compound 5 has been shown to induce autophagic and mitophagic cell death in glioblastoma cells through SIRT1 activation (Yao et al., 2018). Indirect evidence also suggests that SRT1720, SRT2183, and SRT1460, as activators of SIRT1, may modulate autophagy to initiate cancer cell death pathways (Pacholec et al., 2010). Abrus agglutinin, another SIRT1 activator, mediates lipophagy leading to apoptotic cell death through ROS production induced by free fatty acids (Panda et al., 2020). Increased SIRT1 expression is associated with the onset of carcinogenesis and malignant transformation, making SIRT1 inhibition a potential therapeutic strategy. The SIRT1 inhibitor EX527, for example, can acetylate p53 in the presence of etoposide (Solomon et al., 2006), potentially triggering apoptotic cell death and inhibiting protective autophagy (Brooks and Gu, 2008). Despite its mixed results in cancer therapy, EX527 has progressed to phase three clinical trials for Huntington’s disease. The combination of chemotherapy with other SIRT1 inhibitors, such as suramins, JGB1741, tenovins, salermide, sirtinol, and other class III HDAC inhibitors, might enhance the efficacy of cancer treatments by regulating autophagy and inducing associated cell death (Lin and Fang, 2013; Heltweg et al., 2006; Lara et al., 2009; Kalle et al., 2010; Lain et al., 2008; Asaka et al., 2015). The latest inhibitor, MHY2245, affects PKM2/mTOR signaling in ovarian cancer cells, promoting autophagy alongside cell cycle arrest in the G2/M phase and potentially initiating autophagy-associated cell death (Table 4) (Tae et al., 2020).

**TABLE 4:** The regulation of autophagy by SIRT1 in cancer.

Targets	Highlights	References
CDK9	The downregulation of CDK9 suppresses PINK1/PRKN-induced mitophagy to promote mitochondrial dysfunction in hepatocellular carcinoma	Yao et al. (2022)
UBE2C	UBE2C increases SIRT1 ubiquitination to suppress autophagy in endometrial cancer	Zhao et al. (2023e)
SIRT1/AMPK	Quercetin stimulates SIRT1/AMPK axis to mediate autophagy-induced apoptosis	Guo et al. (2021)
SIRT1/FoxO1/Rab7	SIRT1 increases Rab7 expression to induce autophagy in gastric cancer	Zhu et al. (2023b)
SIRT1	SIRT1 stimulates the Beclin-1/autophagy axis in cisplatin resistance in bladder tumor	Sun et al. (2023)
SIRT1	Downregulation of SIRT1 induces autophagy-mediated radiosensitivity in prostate cancer	Wang et al. (2022)
miR-34a	miR-34a is upregulated by catalpol to suppress SIRT1/autophagy in colorectal cancer treatment	Qiao et al. (2020)
Ube2v1	Ube2v1 increases SIRT1 degradation to enhance metastasis of colorectal cancer by autophagy inhibition	Shen et al. (2018)
SIRT/HSPA5	SIRT1/2 downregulation promotes HSPA5 acetylation and mediates protective autophagy in lung cancer	Mu et al. (2019)
miR-138/SIRT1	miR-138 suppresses SIRT1 to inhibit growth, invasion, and autophagy	Ye et al. (2017)
SIRT1	SIRT1 inhibition increases ULK1 acetylation to promote ROS-induced autophagy in colon cancer	Islam et al. (2024b)
miR-124 miR-142	miR-124 and miR-142 downregulate SIRT1 to increase cisplatin sensitivity by autophagy inhibition in lung cancer	Song et al. (2019)
H19/SIRT1	LncRNA H19 stimulates the SIRT1/autophagy axis to induce drug resistance in colorectal cancer	Wang et al. (2018)

A chemical known as silybin has been shown to inhibit SIRT1 and increase p53 acetylation, in addition to its anticancer properties (Yousafzai et al., 2021). Moreover, silybin and the SIRT1 inhibitor cambinol were produced in mice and employed for *in vitro* research according to dosage and time dependent parameters. When it comes to lung adenocarcinoma, silybin has been demonstrated to be an efficient inhibitor of adenocarcinoma, and it has the potential to be utilized as a therapeutic intervention (Liang Z. et al., 2014). HDACs inhibitor tenovin-6 induces apoptosis, suppresses cell migration and invasion, and eliminates cancer stem cells (CSCs) in uveal melanoma (Dai et al., 2016). The progression of uveal melanoma (UM) and the diagnosis have remained pitiful. Tenovin-6 has all of these effects. Inducing a senescence-like growth arrest, perhaps having anticancer potential, and causing an impairment in the activation of the Ras/MAPK pathway are all outcomes of sirtinol, which is another inhibitor. Despite this, sirtinol was found to have an influence on the activation of Akt/PKB as well as the tyrosine phosphorylation of receptors for EGF and IGF-I on the receptors (Ota et al., 2006). On the other hand, SIRT1 suppression by EX527 dramatically decreased the tumor growth of HEC1B and HHUA endometrial cancer. This was due to the fact that SIRT1 overexpression caused cisplatin resistance in HHUA cells, which in turn accelerated carcinogenesis in nude mice. In the treatment of cisplatin-resistant cancer, a combination of EX527 and cisplatin has the potential to be an effective targeted therapy (Asaka et al., 2015). According to computational docking studies, EX527 is solely specific for SIRT1 rather than other sirtuin members. However, Sirtinol, Nicotinamide, and Salermide are all direct targets of inhibitors SIRT1 and 2, and they all have the specific inhibitory action for SIRT1. Salermide is also a direct target of SIRT2. EX527 enhanced carcinogenesis in SCID mice in comparison to the control group, regardless of whether it induces apoptosis and DNA damage *in vitro* (Oon et al., 2015). This suggests that the current method to inhibiting SIRT1 by EX527 *in vitro* and *in vivo* both pancreatic tumor models is unexpectedly the opposite of what was seen *in vitro*. In addition, a study that used short interfering RNA to target SIRT1 found that knocking down SIRT1 can result in the death of cells in the MCF-7 patient line (Peck et al., 2010). MiR-29c overexpression in cisplatin-resistant cancer cells was shown to directly target SIRT1 mRNA and suppress SIRT1 expression. This was demonstrated by Zhang et al. to regulate cell progression and apoptosis, as well as to restore chemosensitivity to cisplatin (Zhang and Luo, 2018). MiR-34a mediated SIRT1 suppression mediates apoptotic activation and chemosensitivity (Herbert et al., 2014). In addition, it is believed that SIRT1 is responsible for accelerating cell growth. In the study of SIRT1’s cellular processes in colorectal cancer, clinical data and patient samples were combined, and a mechanical technique was discovered to regulate p53 and FRA-1 via SIRT1. This approach was verified to be directly related with EMT (Cheng et al., 2016).

## 14 Function of SIRT1 as biomarker

In terms of genetic and epigenetic background, dietary habits, and environmental influences, it has been demonstrated that there are substantial disparities between the populations of Asians and Caucasians (Hur et al., 2008; Tarabay et al., 2016). Not only are these elements necessary for the beginning and advancement of cancer, but they are also necessary for the spread of cancer to other parts of the body (Chatterjee et al., 2018; Pavlidis and Pavlidis, 2018). Mutations and widespread polymorphisms of SIRT1 were discovered in cancer lines produced by Chinese and Japanese individuals (Shimoyama et al., 2011; Shimoyama et al., 2012; Chen et al., 2015; Lv et al., 2017) as well as 41 cancer lines (Han J. et al., 2013). We suggest that differences in SIRT1 mutations and polymorphisms may be one of the causes for differences in predicting OS and TNM stage and lymphatic metastasis of cancer on the basis of SIRT1 expression. This is despite the fact that the data on SIRT1 mutations and polymorphisms are extremely uncommon. It is important to conduct further research on this (Mei et al., 2016). It is well knowledge that metastasis is a factor that may be used to independently predict a bad prognosis for a variety of cancer types (Tsutsumi et al., 2012; Funazo et al., 2017; Ambe et al., 2018). There was a correlation between the higher expression of SIRT1 and OS, DFS, EFS, and PFS. There is a correlation between SIRT1 overexpression and TNM stage, lymph node metastasis, and distant metastasis (Sun et al., 2019); however, there is no correlation with tumor size, tissue invasion depth, differentiation, gender, or age. The overexpression of SIRT1 was found to be predictive with a worse overall survival, as well as a higher TNM stage and lymphatic metastases, in the Asian population, particularly in China. Consequently, the overexpression of SIRT1 may lead to lymphatic metastasis of malignancies, which in turn results in poor overall survival, disease-free survival, event-free survival, and progression-free survival statistics. One of the possible underlying mechanisms for metastasis is the presence of molecular events and biological processes that are mediated by SIRT1. The results of our meta-analysis are in agreement with the findings of SIRT1 being upregulated more frequently in T3 + T4, lymph node metastases, and TNM stage of colorectal cancer patients (Jiang et al., 2014). SIRT1 expression was not connected with these clinicopathological aspects, but rather a poor predictive biomarker of colorectal cancer patients (Byles et al., 2012). This is despite the fact that SIRT1 over-expression was proven to be associated with distant metastasis and histological grade (Jang S-H. et al., 2012). There was a propensity for a high SIRT1 expression to be related with positive lymph node metastasis, despite the fact that a study did not find any significant differences in lymph node metastasis compared to other studies (Otsuka et al., 2022). A high expression of SIRT1 was shown to be strongly linked with lymph node metastasis, according to the findings of two studies that were included in this comparative analysis. In breast cancer (Wu et al., 2012) and colorectal cancer (Zu et al., 2016), there has been reported to be a connection between SIRT1 expression and lymph node metastasis. Furthermore, it has been revealed that SIRT1 expression is implicated in cell migration in prostate cancer (Byles et al., 2012) and non-small-cell lung cancer (Han L. et al., 2013).

SIRT1, as a key regulator of cellular processes such as DNA repair, apoptosis, autophagy, and metabolism, has become a potential therapeutic target in cancer therapy, where both SIRT1 inducers and inhibitors are being explored for different cancer contexts. SIRT1 inducers are of particular interest in cancers where SIRT1 functions as a tumor suppressor. In many cancers, SIRT1 activation promotes genomic stability and DNA repair by deacetylating important regulators such as p53 and FOXO proteins, thereby reducing the accumulation of DNA damage. This function helps prevent oncogenesis by preserving the integrity of the genome. Inducers of SIRT1, such as resveratrol and other small molecules, have been shown to activate SIRT1’s deacetylase activity, which leads to the suppression of tumor progression through the inhibition of cancer cell proliferation and the promotion of apoptosis. Resveratrol, a naturally occurring polyphenol, has garnered attention for its ability to activate SIRT1 and its subsequent anti-cancer effects, particularly in cancers like breast and prostate cancer, where SIRT1’s tumor-suppressive role has been documented. In addition to promoting apoptosis, SIRT1 activation also stimulates autophagy, a process that allows cancer cells to degrade damaged organelles and proteins, thus reducing oxidative stress and promoting cell survival under stress conditions. This duality makes SIRT1 inducers promising for cancers where oxidative stress plays a significant role, offering a cytoprotective effect in normal tissues while targeting cancerous growth. On the other hand, SIRT1 inhibitors are being explored in cancer types where SIRT1 acts as a tumor promoter, particularly in cases of drug resistance and aggressive cancers. For example, in cancers such as hepatocellular carcinoma, pancreatic cancer, and some forms of leukemia, SIRT1 is often upregulated, which leads to enhanced survival of cancer cells through the suppression of apoptosis and the activation of pro-survival pathways. In such cases, inhibiting SIRT1 can restore the cell’s sensitivity to apoptosis-inducing therapies. SIRT1 inhibitors, such as EX527 and nicotinamide, have been shown to enhance the effectiveness of chemotherapeutic agents like cisplatin by increasing the acetylation and activity of pro-apoptotic factors such as p53. By preventing SIRT1 from deacetylating key regulators of apoptosis and cell death, these inhibitors can sensitize cancer cells to treatment, overcoming resistance and leading to more effective cancer eradication. Additionally, SIRT1 inhibitors may interfere with the autophagic survival pathways, further increasing cancer cell susceptibility to stress and cytotoxicity. However, the use of SIRT1 inhibitors must be approached cautiously, as prolonged inhibition of SIRT1 can disrupt normal cellular homeostasis, potentially leading to adverse effects such as metabolic dysregulation or damage to normal tissues. Therefore, identifying the cancer-specific roles of SIRT1 and tailoring the application of its inducers and inhibitors is critical for developing precise and effective cancer therapies.

## 15 Conclusion and future perspectives

The sirtuin family, particularly SIRT1, plays a crucial role in regulating cellular and biological processes. While SIRT1 is essential for normal physiological functions, its dysregulation has been linked to the pathogenesis of various diseases, including cancer. Recent studies have shown that SIRT1 is dysregulated in multiple tumor types, including brain, gastrointestinal, gynecological, and reproductive tumors. Given SIRT1’s influence on numerous pathways and its regulation by diverse upstream mediators, it is critical to delineate the specific mechanisms through which SIRT1 modulates tumorigenesis. Additionally, autophagy, a process extending beyond cell death, has been recognized for its role in tumor cell behavior, impacting cell death, growth, viability, metastasis, and therapy resistance. This review focuses on the interaction between autophagy and SIRT1 in regulating tumorigenesis. Notably, while autophagy generally contributes to protein degradation, it can specifically regulate SIRT1 by targeting it for degradation, thereby suppressing its activity. However, most research has concentrated on how SIRT1 regulates autophagy, with findings that SIRT1 can activate autophagy, including specialized forms like mitophagy and lipophagy. Such regulation can contribute to drug resistance in cancer. The impact of SIRT1-mediated autophagy on cancer drug resistance is yet to be thoroughly investigated across different cancer types and with various chemotherapeutic agents, including topoisomerases. Moreover, SIRT1’s regulation of autophagy often involves major autophagy regulators such as AMPK and mTOR. Intriguingly, SIRT1-mediated autophagy can influence apoptosis in cancer cells; for example, SIRT1-induced pro-survival autophagy can decrease apoptosis, whereas toxic autophagy can enhance it. Despite the development of several SIRT1 regulators, their direct effects on autophagy have not been extensively studied. Future research should focus on drug discovery and the development of small molecules that target SIRT1 to modulate autophagy in cancer treatment.

SIRT1 plays a dual role in cancer, acting as both a tumor suppressor and a tumor promoter depending on the cellular context. As a tumor suppressor, SIRT1 deacetylates and activates key regulatory proteins such as p53, FOXO transcription factors, and RB, which are involved in cell cycle arrest, DNA repair, and apoptosis. This promotes cellular homeostasis and reduces the likelihood of oncogenic transformation. Additionally, SIRT1’s role in maintaining genomic stability and preventing oxidative stress further supports its tumor-suppressive functions, particularly in early stages of cancer development. In various cancer types, SIRT1 overexpression has been linked to reduced tumorigenicity and enhanced sensitivity to chemotherapy. Conversely, SIRT1 can also act as a tumor promoter, particularly in advanced cancers, where it aids in tumor progression by promoting cell survival and resistance to stress. SIRT1 has been shown to inhibit apoptosis by deacetylating and inactivating pro-apoptotic factors, such as p53 and E2F1, leading to enhanced tumor cell survival. It also contributes to the activation of oncogenic pathways, including those involving NF-κB and MYC, which drive cancer cell proliferation and metastasis. Moreover, SIRT1 has been implicated in promoting drug resistance by modulating autophagy and DNA repair pathways, making tumors more resilient to conventional therapies. This dual nature of SIRT1 highlights the importance of context in determining its role in cancer progression.

SIRT’s role in autophagy is tightly linked to several key molecular pathways, such as the mTOR (mechanistic target of rapamycin) and AMPK (AMP-activated protein kinase) pathways. SIRT1 influences these pathways in ways that either promote or regulate autophagy, depending on the cellular context. SIRT1 activates autophagy primarily by deacetylating various proteins involved in the autophagic machinery, such as ATG5, ATG7, and ATG8, and also deacetylates the transcription factor FOXO3, promoting the expression of autophagy-related genes, including LC3. In the mTOR pathway, a major negative regulator of autophagy, SIRT1 indirectly inhibits mTOR, promoting autophagy. This inhibition occurs through the activation of TSC1/2, a negative regulator of mTORC1, and by activating AMPK, which enhances the inhibition of mTORC1. Under nutrient-rich conditions, mTOR suppresses autophagy by preventing autophagosome formation, but SIRT1-mediated inhibition of mTOR reverses this effect. On the other hand, SIRT1 activates AMPK by deacetylating liver kinase B1 (LKB1), which leads to the phosphorylation of TSC2 and RAPTOR, thus promoting autophagy. Activated AMPK also directly phosphorylates ULK1, an initiator of autophagy. This interaction between SIRT1 and AMPK is critical in energy-deficient states, allowing cells to initiate autophagy to survive under stress. SIRT1 also affects autophagy through its interaction with p53, a tumor suppressor that inhibits autophagy when acetylated. By deacetylating and inactivating cytoplasmic p53, SIRT1 reduces its inhibitory effects on autophagy. Furthermore, SIRT1 modulates FOXO transcription factors, particularly FOXO1 and FOXO3, which promote the expression of autophagy-related genes when deacetylated by SIRT1. This enhances autophagic processes, especially during stress conditions. Additionally, SIRT1 influences mitochondrial autophagy (mitophagy) by deacetylating and activating PGC-1α, a key regulator of mitochondrial biogenesis and energy metabolism. Another important autophagy regulator influenced by SIRT1 is Beclin-1, a key protein in autophagosome formation. SIRT1 interacts with and enhances the activity of Beclin-1, further promoting autophagy. Moreover, SIRT1 affects multiple molecular pathways, such as its interaction with mTOR and AMPK, highlighting its central role in coordinating cellular energy homeostasis and stress responses. By integrating signals from various pathways, including mTOR, AMPK, FOXO, and p53, SIRT1 balances cell survival and degradation under stress conditions. These mechanisms demonstrate the significant role of SIRT1 in promoting autophagy, making it a crucial factor in cellular health, energy regulation, and potential therapeutic targets for diseases linked to autophagy dysfunction.

As a NAD + -dependent deacetylase, SIRT1 influences cancer cell survival by modulating stress responses, DNA repair, and the tumor microenvironment, contributing to the development of resistance to chemotherapy and targeted therapies. Understanding the relationship between SIRT1 and drug resistance, particularly through autophagy, is essential to developing effective therapeutic strategies. SIRT1 contributes to drug resistance in multiple cancer types by promoting cancer cell survival under stress. It deacetylates and activates various transcription factors, such as p53, FOXO, and NF-ĸB, which are involved in cellular stress responses and apoptosis. Through these interactions, SIRT1 enhances the ability of cancer cells to withstand chemotherapeutic agents and resist apoptosis. For example, in breast cancer, SIRT1 has been shown to deacetylate and inhibit p53, a tumor suppressor, allowing cancer cells to escape apoptosis induced by DNA-damaging agents. Additionally, in hepatocellular carcinoma (HCC), SIRT1-mediated pathways are associated with resistance to sorafenib, a common drug used in HCC treatment. Autophagy is a cellular degradation process that plays a dual role in cancer, acting as a tumor suppressor in early stages and a survival mechanism in advanced cancers. SIRT1 is a key regulator of autophagy, particularly under conditions of stress, such as nutrient deprivation or chemotherapy. By deacetylating autophagy-related proteins (ATGs) and transcription factors like FOXO1/FOXO3, SIRT1 promotes the formation of autophagosomes and enhances the autophagic flux, allowing cancer cells to recycle cellular components and sustain energy production during chemotherapy-induced stress. The SIRT1-autophagy axis has been implicated in drug resistance across various cancer types. For instance, in colorectal cancer, SIRT1 activation enhances autophagy, which protects cancer cells from chemotherapy-induced apoptosis by degrading damaged organelles and proteins. In drug-resistant esophageal cancer cells, SIRT1 upregulation has been linked to increased autophagy, leading to enhanced cell survival and resistance to chemotherapy. In these cases, inhibition of SIRT1 or autophagy sensitizes cancer cells to chemotherapy, indicating the pivotal role of the SIRT1-autophagy pathway in mediating drug resistance. SIRT1 promotes autophagy by deacetylating key regulators of the autophagic process, such as Beclin-1 and LC3. It also regulates autophagy-related miRNAs, including miR-34a and miR-215, which affect the expression of autophagy proteins like Atg14. Furthermore, SIRT1 inhibits mTOR (mechanistic target of rapamycin), a negative regulator of autophagy, through pathways involving AMPK activation, thus promoting autophagy under stress conditions. This activation of autophagy by SIRT1 enables cancer cells to maintain cellular homeostasis and evade the cytotoxic effects of chemotherapy. In drug-resistant cancer cells, increased SIRT1-mediated autophagy allows the cells to clear damaged components and maintain survival despite the presence of chemotherapeutic agents. For example, in ovarian cancer, SIRT1-mediated autophagy has been shown to contribute to resistance to cisplatin, while in gastric cancer, SIRT1 enhances autophagy to protect cancer cells from apoptosis induced by 5-fluorouracil. Blocking SIRT1 or inhibiting autophagy in these models reverses drug resistance, further highlighting the importance of this pathway in maintaining cancer cell survival during treatment.

The research on SIRT1 and its role in autophagy has advanced significantly, but its complexity presents several limitations. One key challenge is the dual role of SIRT1 in cancer, where it can function as both a tumor suppressor and promoter depending on the context. In some cancers, SIRT1 activation supports autophagy and cell survival, while in others, it triggers apoptosis and suppresses tumor growth. The context-specific roles of SIRT1, as well as the dual nature of autophagy, complicate the development of generalized therapeutic strategies. This complexity makes it difficult to predict when SIRT1-mediated autophagy would either aid or hinder treatment, especially given the need to target specific cellular environments in cancer. Another limitation stems from the incomplete understanding of the molecular mechanisms behind SIRT1’s regulation of autophagy. While SIRT1’s interactions with autophagy-related proteins like Beclin-1 and FOXO have been noted, the precise pathways it influences remain unclear. This knowledge gap limits the ability to fully exploit SIRT1 as a therapeutic target. Furthermore, SIRT1 is involved in various cellular pathways, including those regulating metabolism and DNA repair, which complicates its therapeutic targeting. The potential for off-target effects or unwanted consequences from influencing multiple pathways simultaneously represents a significant challenge in developing SIRT1-targeted therapies. Currently available SIRT1 activators and inhibitors lack the specificity needed for effective clinical application. Compounds such as resveratrol and EX527 not only target SIRT1 but also affect other members of the sirtuin family and related pathways, leading to potential side effects. Furthermore, finding the optimal dosage and timing of SIRT1 modulation is challenging because over-activation or inhibition of SIRT1 can either promote survival or induce apoptosis in cancer cells. This delicate balance underscores the need for more selective and precise pharmacological tools to modulate SIRT1 activity in a controlled manner. Translating promising preclinical results into clinical practice has proven difficult, particularly due to differences between animal models and human physiology. Tumor heterogeneity further complicates the development of SIRT1-targeted therapies, as the role of SIRT1 and autophagy can vary not only between different cancer types but also within different regions of the same tumor. Additionally, cancer cells can develop resistance to SIRT1 modulators, limiting the long-term effectiveness of these treatments. Understanding and overcoming these resistance mechanisms will be essential for successful clinical application. Finally, the long-term safety of SIRT1-targeted therapies remains uncertain. SIRT1 is involved in many critical cellular processes, including aging and DNA repair, so long-term inhibition or activation could have adverse effects, such as metabolic disorders or neurodegenerative diseases. Moreover, reliable biomarkers to predict patient response to SIRT1-targeted therapies are lacking, making it difficult to assess which patients would benefit most from these treatments. Addressing these limitations will be crucial to advancing SIRT1-targeted therapies into clinical practice, offering new hope for effective cancer treatments.

Although the function of SIRT1 in the regulation of autophagy was covered in the present review, there are also other members of SIRT family participating in the regulation of autophagy. SIRT2, primarily localized in the cytoplasm, has been shown to regulate autophagy through its deacetylation of key autophagic proteins and its involvement in energy metabolism. SIRT2 can deacetylate FOXO1 and FOXO3, transcription factors that upregulate autophagy-related genes. Additionally, SIRT2 affects autophagy by modulating the acetylation of LC3, a key autophagy marker. By promoting LC3 deacetylation, SIRT2 enhances autophagosome formation and autophagic flux. SIRT2 has also been linked to the regulation of mTOR, a key negative regulator of autophagy. By inhibiting mTOR activity, SIRT2 indirectly promotes autophagy under conditions of nutrient stress. However, SIRT2’s role in autophagy can be complex, as in some contexts, it has been observed to inhibit autophagy and promote cell proliferation, particularly in cancer. SIRT3, SIRT4, and SIRT5 are mitochondrial sirtuins that regulate autophagy through their effects on mitochondrial function and metabolism. SIRT3 is the most well-studied of the three and plays a key role in regulating mitophagy, a specific form of autophagy that targets damaged mitochondria for degradation. SIRT3 deacetylates several mitochondrial proteins, enhancing mitochondrial respiration and reducing oxidative stress, which can influence autophagy activation. In response to cellular stress, SIRT3 can enhance autophagy by deacetylating and activating FOXO3, which upregulates autophagy-related genes such as Beclin-1 and LC3. Additionally, SIRT3 inhibits mTOR signaling by promoting the activation of AMPK, an energy sensor that stimulates autophagy. SIRT4, while less studied, has been shown to inhibit autophagy through its role in regulating mitochondrial glutamine metabolism. By inhibiting glutamate dehydrogenase (GDH), SIRT4 suppresses the production of ATP and thus limits the energy supply needed for autophagy, leading to decreased autophagic activity. SIRT5, a mitochondrial lysine demalonylase and desuccinylase, can also regulate mitochondrial function and oxidative stress, although its direct involvement in autophagy is still under investigation. SIRT6 is primarily a nuclear sirtuin involved in DNA repair and metabolic regulation, but it also influences autophagy. SIRT6 can enhance autophagy by promoting the activation of the AMPK pathway, leading to the inhibition of mTOR, thus stimulating autophagy. Additionally, SIRT6 regulates autophagy by deacetylating histones at the promoters of autophagy-related genes, promoting their transcription. For example, SIRT6-mediated deacetylation of histone H3 at lysine 9 (H3K9) near the promoter region of genes such as ATG5 and ATG12 enhances autophagy induction. SIRT6 also affects the autophagy-lysosomal pathway, which is critical for maintaining cellular homeostasis, particularly during stress. SIRT7, another nuclear sirtuin, has a more indirect role in autophagy regulation. SIRT7 primarily regulates ribosomal RNA (rRNA) transcription and protein synthesis, which affects cellular growth and metabolism. By modulating metabolic pathways, SIRT7 influences the availability of nutrients and energy, which can impact autophagy activation. Interestingly, SIRT7’s suppression of autophagy has been linked to its role in cancer, where it promotes cancer cell survival by limiting autophagic processes. SIRT7 has been observed to deacetylate and inhibit proteins involved in autophagy initiation, thus reducing autophagic flux in certain cancer contexts. More information about SIRT2, SIRT3, SIRT4, SIRT5, SIRT6 and SIRT7 can be found in these reviews (Chen et al., 2020; Wang et al., 2019c; Torrens-Mas et al., 2017; Alhazzazi et al., 2011; Wang et al., 2020; Tomaselli et al., 2020; Bringman-Rodenbarger et al., 2018; Lagunas-Rangel, 2023; Fiorentino et al., 2021; Tang M. et al., 2021).
